# Supplementary material for: Functional assembly of the Qc-SNARE with Sec18 and Sec17 on membranes
Source: Mol Biol Cell. 2025 Dec 19;37(1):ar5. doi: 10.1091/mbc.E25-10-0474 (PMC12766925; doi:10.1091/mbc.E25-10-0474)
Supplement: Supplementary file 1 [file mbc-37-ar5-s001.pdf]

Functional assembly of the Qc-SNARE with Sec18 and Sec17 on membranes

Amy Orr, Karina Lopes, Jerry O'Dwyer, and William Wickner*
Department of Biochemistry and Cell Biology, Geisel School of Medicine at Dartmouth, 7200 Vail Building, Hanover, NH 03755

ABSTRACT Yeast vacuolar fusion is driven by Sec17, Sec18, SNAREs of four families (R [Nyv1], Qa [Vam3], Qb [Vti1], Qc [Vam7]) and HOPS, a catalyst of SNARE assembly. Qc, the only vacuolar SNARE that is not membrane-anchored, has a unique path of assembly with other fusion catalysts. Qc is the only SNARE that binds Sec17 with high affinity. Sec18 confers a high affinity for Qc (but not Qb) on HOPS-dependent fusion, but it has been unclear how Sec18 acts. The membrane complex of Sec17 and Sec18 binds Qc to form a membrane:Sec18:Sec17:Qc complex. Sec18 ATP hydrolysis, though dispensable for fusion, provides a measure of the physical and functional interactions between Qc, Sec17, Sec18, and membranes. Each binary interface in this quaternary complex regulates Sec18 ATPase and fusion. Qc is better than other SNAREs, alone or in combination, for stimulating ATP hydrolysis. We propose a working model in which membrane-bound Qc:Sec17:Sec18 associates with the *trans* complex of HOPS:R:QaQb, displacing HOPS while providing both Qc for complete SNARE zippering and localized Sec17 apolar loops, the twin driving forces for fusion.

Monitoring Editor
Elizabeth Miller
MRC Laboratory of Molecular Biology

Received: Oct 3, 2025
Revised: Nov 10, 2025
Accepted: Nov 13, 2025

SIGNIFICANCE STATEMENT

- The vacuolar Qc SNARE, like many other Qc-SNAREs, lacks an integral membrane anchor and thus requires catalysis of its delivery to the growing *trans*-SNARE complex.
- Sec17 and Sec18 form a membrane-bound complex with Qc, then all three join the nascent *trans*-SNARE complex.
- SNARE assembly is highly regulated by SM proteins, Sec17/SNAP, and Sec18/NSF.

INTRODUCTION

Intracellular membrane fusion on the endocytic and exocytic pathways is catalyzed by conserved proteins, including Rab-family GTPases (Hutagalung and Novick, 2011), Rab-binding complexes to tether membranes (Baker and Hughson, 2015), SNARE proteins anchored in each membrane (Jahn and Scheller, 2006),

This article was published online ahead of print in MBoC in Press (http://www.molbiolcell.org/cgi/doi/10.1091/mbc.E25-10-0474) on November 19, 2025.

Author contributions: A.O. and W.W. conceived and designed the experiments. A.O. and W.W. performed the experiments. A.O. and K.L. analyzed the data. A.O. and W.W. drafted the article. A.O. and K.L. prepared the digital images. K.L. performed recombinant protein design and purification. J.O. provided alpha-fold insights into possible structures of various components.

Conflict of interest: The authors declare no competing financial interests.

*Address correspondence to: William Wickner (Bill.Wickner@Dartmouth.edu).

Abbreviations used: HOPS, Homotypic fusion and vacuole protein sorting; FRET, Fluorescence resonance energy transfer; NSF, N-ethylmaleimide-sensitive factor; RPL, reconstituted proteoliposome; SNAP, Soluble NSF attachment protein; SNARE, Soluble NSF attachment protein receptor; VML, vacuolar mixed lipids.

© 2025 Orr *et al*. This article is distributed by The American Society for Cell Biology under license from the author(s). It is available to the public under an Attribution 4.0 International Creative Commons CC-BY 4.0 License (https://creativecommons.org/licenses/by/4.0/).

"ASCB®," "The American Society for Cell Biology®," and "Molecular Biology of the Cell®" are registered trademarks of The American Society for Cell Biology.

SM (Sec1/Munc18)-family SNARE assembly chaperones (Rizo and Sudhof, 2002), Sec17/SNAP, and the Sec18/NSF AAA-family ATPase (Söllner *et al.*, 1993). Rab GTPases bind their cognate tethering proteins. Tethering allows SNAREs to associate in their parallel, active conformation (Weninger *et al.*, 2003; Zick and Wickner, 2014; Song and Wickner, 2019). SNARE proteins consist of diverse N-domains, 50–60 amino acid SNARE domains with heptad repeat apolar residues, a short juxtamembrane region, and (often) a C-terminal transmembrane anchor. SNAREs are in four conserved families, termed R, Qa, Qb, and Qc according to whether they have a central arginyl or glutaminyl residue instead of their central apolar residue (Fasshauer *et al.*, 1998). Before fusion, the R- and Q-SNAREs are on opposite fusion partner membranes (Fukuda *et al.*, 2000). Their SNARE domains alone are inherently random-coil but become alpha-helical as they progressively associate in the N- to C-direction, termed zippering, to form RQaQbQc 4-helical *trans*-SNARE complexes (Sorensen *et al.*, 2006). Zippering is driven by the energy of additional water hydrogen bonding as the SNARE heptad-repeat hydrophobic amino acyl side-chains are buried in the center of the 4-SNARE bundle. It was suggested that SNAREs simply self-assemble in *trans*, then promote fusion by zippering to draw the two membranes together, and that other proteins only regulate the levels of *trans*-SNARE complex. However, studies with reconstituted human synaptic fusion (Ma *et al.*, 2013; Prinslow *et al.*, 2019; Stepien and Rizo, 2021) and yeast vacuolar fusion (Mima *et al.*, 2008; Zick and Wickner, 2016) show a greater multiplicity of required proteins whose actions do not simply correlate with *trans*-SNARE abundance.

We explore membrane fusion mechanisms through the homotypic fusion of yeast vacuoles (lysosomes). Vacuole fusion has been reconstituted with all purified components (Fukuda *et al.*, 2000; Mima *et al.*, 2008; Stroupe *et al.*, 2009; Zick and Wickner, 2016). The fusion of proteoliposomes of vacuolar mixed lipids (VML) and purified recombinant vacuolar fusion proteins (Mima *et al.*, 2008; Stroupe *et al.*, 2009; Zick and Wickner, 2016) is monitored by a quantitative fluorometric assay (Zucchi and Zick, 2011). Vacuoles and reconstituted proteoliposomes have a GTPase (Ypt7) of the Rab family (Haas *et al.*, 1995), termed simply Rab hereafter. The vacuolar SM protein (Vps33) is a subunit of the hexameric HOPS (homotypic fusion and vacuole protein sorting) protein (Seals *et al.*, 2000; Shvarev *et al.*, 2022), which has Vps 11, 16, 18, 33, 39, and 41 subunits (Wada *et al.*, 1992; Seals *et al.*, 2000). The vacuolar SNAREs (Nichols *et al.*, 1997; Ungermann *et al.*, 1998) are Nyv1 (R), Vam3 (Qa), Vti1 (Qb), and Vam7 (Qc), referred to hereafter as simply R, Qa, Qb, and Qc. Two HOPS subunits, Vps39 and Vps41, have direct affinity for the Rab on each fusion partner membrane, thereby tethering the vacuoles (Brett *et al.*, 2008; Hickey and Wickner, 2010; Brocker *et al.*, 2012). HOPS has direct binding affinity for each of the four SNAREs (Song *et al.*, 2020; Baker *et al.*, 2015), for Sec17 (Song *et al.*, 2021), for Sec18 (Orr and Wickner, 2024), and for phosphoinositides such as PI3P (Stroupe *et al.*, 2006). Parallel grooves on the surface of the SM subunit of HOPS bind the N-terminal end of the R and Qa SNARE domains, positioning them to initiate 4-SNARE assembly (Baker *et al.*, 2015). Early in zippering, the residues of R and Qa which lie in the grooves on the SM HOPS subunit reorient into the center of the nascent 4-helical SNARE complex. This may weaken SNARE:HOPS binding. Sec17 can complete the displacement of HOPS from the *trans*-SNARE complex (Collins *et al.*, 2005; Schwartz *et al.*, 2017), and virtually all the *trans*-SNARE complex on vacuoles is associated with Sec17 (Xu *et al.*, 2010). The pre-fusion structure of SNAREs, Sec17, and Sec18 (Song *et al.*, 2021) is modeled on the 20s complex of synaptic SNAREs, NSF, and SNAP (Zhao *et al.*, 2015), but with the SNAREs anchored in *trans*. In this structure, several Sec17/SNAP molecules form a sheath around the 4-SNARE bundle, with the N-terminal apolar loop of each Sec17/SNAP engaging membrane lipids, with ionic bonds between neighboring Sec17 molecules in the sheath (Ma *et al.*, 2016) and between Sec17 and residues on the surface of the 4-SNARE *trans*-complex, and with the Sec17 C-terminal leucyl residues bound to the large Sec18 hexamer. Two models have been envisioned for the assembly of this structure. In one model, the SNAREs first assemble, then several Sec17 molecules bind to the assembled SNAREs and serve as a receptor to bind Sec18. In this model, Sec18 would have no effect on the EC50 of Sec17 for fusion. A more recent model (Orr and Wickner, 2022) is that several Sec17 molecules bind reversibly to a Sec18 hexamer as a 3Sec17:Sec18 complex, which stably associates with membranes by the product of the weak affinities of the N-domain apolar loop of each Sec17 for lipid. After the interdependent membrane binding of Sec17 and Sec18, their complex binds to SNAREs. This model was suggested by the findings that fusion without complete SNARE zippering is highly cooperative for Sec17 concentration in the absence of Sec18 but not in its presence (Orr and Wickner, 2022), suggesting that Sec18 helps gather multiple Sec17s, and that Sec17 and Sec18 are interdependent for their stable membrane association (Orr and Wickner, 2022; Lopes *et al.*, 2025). In accord with these findings, we now report that Sec17 and liposomes cooperate to enhance ATP hydrolysis by Sec18 in the absence of SNAREs.

Though Sec18 ATPase and Sec17 act after membrane fusion, using energy from ATP binding and hydrolysis to drive the disassembly of *cis*-SNARE complex (Söllner *et al.*, 1993; Haas and Wickner, 1996), Sec17 and Sec18 also act before fusion. Schwartz and Merz (2009) showed that C-terminal truncation of the Qc SNARE domain blocks fusion in a manner that is bypassed by added Sec17 and further stimulated by Sec18 (Zick *et al.*, 2015). Sec17 and Sec18 also bypass fusion blockade from any of several causes: C-terminal truncation of any of the Q- SNAREs (Song *et al.*, 2021), swap of the juxtamembrane domains between R and Qa (Orr *et al.*, 2022), having Qb as the sole membrane anchored Q-SNARE (Wickner *et al.*, 2023), or replacement of the Qa heptad repeat apolar residues with Gly, Ala, or Ser (Song *et al.*, 2021). Even with wild-type SNAREs and unimpeded zippering, Sec17 and Sec18 restore fusion which had been impeded by stiff fatty acyl lipid composition (Song *et al.*, 2024), and Sec18 is essential for fusion with limiting HOPS levels (Orr and Wickner, 2024). Sec17 and Sec18 also stimulate fusion under even the most favorable fusion conditions (Song *et al.*, 2017). Slow fusion can be driven by either SNARE zippering (without Sec17 or Sec18) or by Sec17 and Sec18 (without energy from completion of SNARE zippering), but rapid fusion needs both. The apolarity of the small loop in the Sec17 membrane-proximal N-domain is critical for Sec17 promotion of fusion (Song *et al.*, 2021) and may act as a wedge into the tightly apposed bilayers.

Sec18 can lower the EC50 for the Qc-SNARE for fusion as much as 100-fold (Orr and Wickner, 2024), yet has no effect on the EC50 for the Qb-SNARE. The mechanism of this SNARE-specific Sec18 action has been unclear. We now report that Qc associates with Sec17 and Sec18 on membranes. Though ATP hydrolysis is not needed for membrane fusion (Zick *et al.*, 2015), ATP hydrolysis by Sec18 is a sensitive measure of Sec18 associations with SNAREs, Sec17, and membranes (Barnard *et al.*, 1997; Winter *et al.*, 2009; Cipriano *et al.*, 2013; Barnard *et al.*, 2017). We find that this Sec18 activation does not require classical RQaQbQc bundles, anchored

in *trans* or in *cis*, but needs only Qc, Sec17, and membrane lipid. PI3P localizes Qc to the membrane surface, where it can bind the Sec17:Sec18 complex. Sec18:Sec17:Qc:membrane association, assayed both physically and by Sec18 catalytic activation, is promoted by specific associations: the binding of the Qc PX domain to phosphatidylinositol-3-phosphate (PI3P) at the membrane (Cheever *et al.*, 2001), the recognition of the Qc SNARE domain by Sec17, Sec17 binding to membranes by its N-domain apolar loop (Winter *et al.*, 2009; Zick *et al.*, 2015), and Sec17 binding Sec18 by its C-terminal leucyl residues (Schwartz and Merz, 2009; Barnard *et al.*, 2017). This Qc activation of Sec18 is in accord with the specific association of the Qc SNARE domain in the synaptic SNARE complex with NSF (White *et al.*, 2018). These same interactions between Qc, membranes, Sec17, and Sec18 stimulate membrane fusion. Sec18 may lower the EC50 of Qc for fusion (Orr and Wickner, 2024) by delivering Qc and Sec17 to the rest of the membrane fusion apparatus.

RESULTS

Binding relationships between Sec17, Sec18, SNAREs, and liposomes

To explore SNARE associations, proteoliposomes without proteins or bearing one or several anchored SNAREs were incubated with Sec17, then recovered after floatation up a density gradient and analyzed by immunoblot for bound Sec17. The low levels of Sec17 bound to proteoliposomes with R, Qa, or Qb were comparable with the Sec17 bound to liposomes that bore no protein (Figure 1A, lanes 1–4). Substantially more Sec17 bound to proteoliposomes with Qc anchored to membranes by a recombinant C-terminal transmembrane segment, termed Qc-tm (Figure 1A, lane 5). Liposomes bearing several SNAREs allowed Sec17 binding (Figure 1A, lanes 6–10), even without Qc (lanes 6, 8, and 9), but the addition of Qc gave substantial binding increases (lanes 6 vs 7, and 9 vs 10).

Does Sec17 bind Qc by forming an oligomeric complex of several Sec17 molecules surrounding a coiled coil of several Qc molecules, much as several Sec17 molecules surround RQaQbQc in the 20s structure (Zhao *et al.*, 2015)? When Sec17 is oligomerized, either by association with four SNAREs without their transmembrane anchors, termed soluble-SNAREs (Zick *et al.*, 2015), or with hexameric Sec18 (Orr and Wickner, 2022), it binds membranes with the product of the weak lipid affinities of each of the several Sec17 molecules, converting a very weak lipid affinity of the Sec17 monomer to a very strong liposome affinity of any complex which includes several Sec17 molecules. Neither Qc nor the soluble domains of any of the other three SNAREs, sR, sQa, or sQb, induces Sec17 to oligomerize and thereby bind to liposomes (Figure 1B, lanes 1–5). Sec17 binds well when it can associate with the three soluble Q-SNAREs (Figure 1B, lane 6) or all four soluble SNAREs (lane 7). These findings suggest that Qc alone does not form an oligomer for Sec17 binding.

Qc binds to membranes with PI3P (Figure 2, lane 1), and Sec17 binds weakly by its apolar loop to membranes (lane 2). The membrane binding of each is unaltered by the presence of the other (lane 3). Sec18 binds to membranes (lane 4) by its affinity for phosphatidic acid (Starr *et al.*, 2016). This Sec18 binding is unaffected by Qc (lane 5). A 3Sec17:Sec18 complex (Orr and Wickner, 2022) strongly enhances both Sec17 and Sec18 membrane binding (lane 6 vs 2 and 4). The 3Sec17:Sec18 complex binds additional Qc to membranes (lane 7 vs 1, 3, 5). Since membranes are known to enhance NSF and SNAP-dependent SNARE complex disassembly (Winter *et al.*, 2009), we asked whether Qc without the other SNAREs could stimulate Sec18 ATPase when it associates with lipid, Sec17, and Sec18.

Synergy among factors stimulating Sec18 ATPase. We find that Sec18 ATP hydrolysis is exquisitely regulated by interactions between Sec18, Sec17, the Qc-SNARE, and membranes, as illustrated in the scheme of Figure 3A. The number of Qc and Sec17, which associate at the membrane with each other and with Sec18, is not known, but for simplicity, only one of each is drawn. There are several direct binding interactions in this scheme: the Qc N-terminal PX domain with its membrane lipid receptor PI3P (Cheever *et al.*, 2001), the N-terminal region of the Qc SNARE domain with Sec18 (White *et al.*, 2018), the SNARE domain of Qc with Sec17 (Marz *et al.*, 2003; Zhao *et al.*, 2015), the N-domain apolar loop of Sec17 with the lipid bilayer (Winter *et al.*, 2009; Zick *et al.*, 2015), and the C-terminal leucyl residues of Sec17 with Sec18 (Barnard *et al.*, 1997; Schwartz and Merz, 2009; Barnard *et al.*, 2017). We asked whether each regulates ATP hydrolysis by Sec18 (Figure 3, B and C; *t* tests of significance are shown in parentheses in the text for Figure 3B for clariry). Sec18 hydrolysis of ATP (Söllner *et al.*, 1993) showed only slight stimulation by either Sec17 (Figure 3B, lanes 11 vs 8*) as reported (Morgan *et al.*, 1994) or by protein-free liposomes of vacuolar lipid composition (lanes 9 vs 8*) and was not stimulated by Qc SNARE alone (lanes 10 vs 8, ns). Combinations of Sec18, Sec17, liposomes, and Qc caused synergistic stimulations. There was greater stimulation of Sec18 ATP hydrolysis by Sec17 and liposomes (lane 14) than by either Sec17 (lane 14 vs 11***) or liposomes (lane 14 vs 9***) alone. This relied on the Sec17 C-terminal leucyl residues, which bind Sec18 (Barnard *et al.*, 1997; Schwartz and Merz, 2009; Bernard *et al.*, 2017). These residues are substituted in Sec17L291AL292A, termed Sec17LALA (lane 16, vs wild-type Sec17 in lane 14***). Stimulation also relied on the Sec17 N-domain loop apolarity for binding membranes (Winter *et al.*, 2009; Zick *et al.*, 2015), substituted in Sec17F21SM22S, termed Sec17FSMS (lane 17, vs wild-type in lane 14***). We have previously reported two independent assays of the interactions of Sec17 with both membranes and Sec18. In functional assays of fusion with blocked SNARE zippering, Sec18 is required for fusion at limiting Sec17 (Orr and Wickner, 2022). In a physical assay of association, Sec17 and Sec18 exhibited interdependent binding to protein-free liposomes in floatation assays (ibid, and Figure 2). The finding that lipids and Sec17 strongly stimulate the ATPase activity of Sec18, and that this stimulation needs the Sec17 binding sites for lipid and Sec18, provides a third and independent indication of Sec18:Sec17:lipid interactions.

The further presence of Qc-SNARE causes substantially greater stimulation of Sec18 ATP hydrolysis (Figure 3B, lane 18). This ATPase activity relies on Sec17 (lanes 18 vs 13***), Qc (lanes 18 vs 14***), and liposomes (lanes 18 vs 12***). PI3P, a membrane receptor for Qc (Cheever *et al.*, 2001), was needed for Qc stimulation of Sec18 ATPase in the presence of Sec17 and Qc (lanes 18 vs 20***), though PI3P omission did not affect ATP hydrolysis without Qc (lanes 14 vs 15, ns). The Y42A mutation in the Qc PX domain, which ablates the Qc affinity for PI3P (Cheever *et al.*, 2001), reduced its stimulation of Sec18 ATPase (lanes 18 vs 23***). In contrast, Qc stimulation of Sec18 ATPase in the presence of lipids and Sec17 does not require the C-terminal portion of the SNARE domain (lane 18 vs 19 ns). Proteins that contribute to optimal Sec18 ATPase have direct membrane affinity: Sec17 by its N-domain apolar loop, which is ablated by the FSMS mutation, and Qc by the affinity of its PX domain for PI3P, which is lost without this lipid

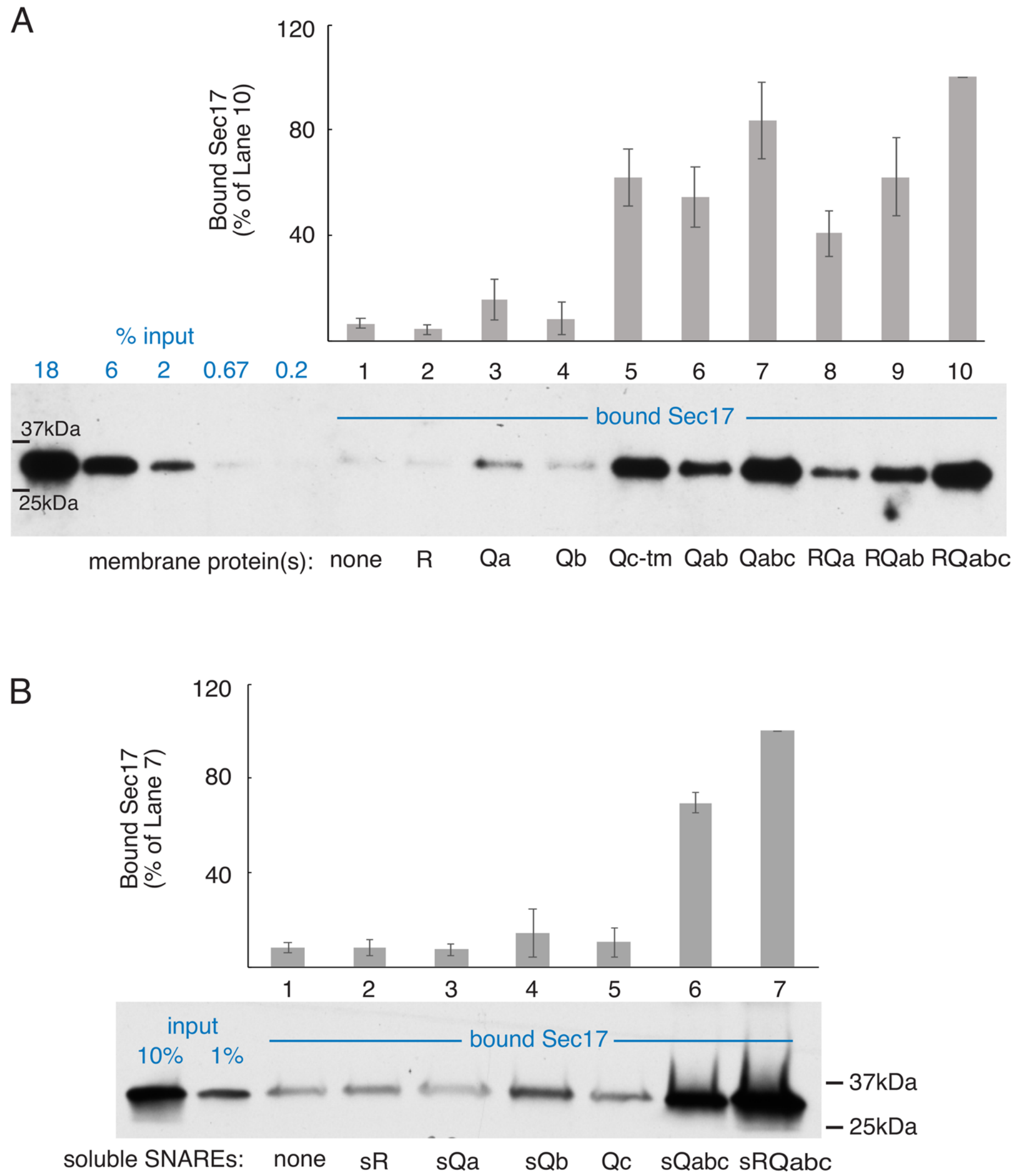

FIGURE 1: Sec17 binds preferentially to membrane-bound Qc. VML proteoliposomes were prepared for binding assays as described in *Materials and Methods* with no SNAREs, single membrane bound SNAREs, or combinations of the wild-type SNAREs as indicated. Binding assays were conducted as described in *Materials and Methods* with (A) 1 μM added Sec17 or (B) protein-free VML liposomes, 2 μM Sec17 and 2 μM soluble SNAREs as indicated. Averages and standard deviations of triplicate experiments are shown.

or when Qc binding was inactivated by the Y42A mutation. Sec18 binds membranes by its affinity for phosphatidic acid (Starr *et al.*, 2016) as well as through its bound Sec17 (Orr and Wickner, 2022).

Sec17 binds Sec18 by the Sec17 C-terminal leucyl residues and binds membranes by the Sec17 apolar loop (Winter *et al.*, 2009; Zick *et al.*, 2015; Orr and Wickner, 2022). Qc may engage the Sec17:Sec18 complex by direct affinity of its SNARE domain for Sec17 (Zhou *et al.*, 2015; also refer to Figures 1, 3C, and [below] 5) and for Sec18 (White *et al.*, 2018), while the Qc N-terminal PX domain engages membrane PI3P (Cheever *et al.*, 2001). The assembly of membrane, Qc, Sec17, and Sec18 has at least two functional redundancies for stimulating Sec18. There is more ATP hydrolysis when Qc binding to membrane PI3P is lost through the Y42A mutation than when Qc is altogether absent (Figure 3B, lane 23 vs 14*), suggesting that Qc can functionally engage through only binding Sec17 and Sec18. Similarly, when the Sec17 binding to Sec18 is weakened by the Sec17LALA mutation, Sec18 ATP hydrolysis is reduced but remains greater than seen without Sec17 (Figure 3B, lane 21 vs 13***) or without Qc (lane 21 vs 16***).

Sec17 is adjacent to the SNARE domains of Qc and the other SNAREs in the classic 20s structure (Zhao *et al.*, 2015). The ionic bonds between Sec17 and SNARE domains are weakened by substitution of acidic amino acyl residues for basic lysyl residues in the Sec17 mutant Sec17K159EK163E (Marz *et al.*, 2003, Schwartz *et al.*, 2017), termed Sec17KEKE. Sec17KEKE retains its overall structure since, in the absence of Qc, Sec17KEKE stimulation of Sec18 ATP hydrolysis is comparable to that from wild-type Sec17 (Figure 3C, lane 4 vs 5). In the presence of Qc, Sec17KEKE stimulates less than wild-type Sec17 (lanes 7 vs 8), though more than seen in the absence of Sec17 (lanes 8 vs 6). Though the Rab Ypt7 is required for fusion of vacuoles (Haas *et al.*, 1995; Seeley *et al.*, 2002) or of vacuole-derived proteoliposomes (Mima and Wickner, 2009; Stroupe *et al.*, 2009), it does not affect the hydrolysis of ATP by Sec18 (Supplemental Figure S1).

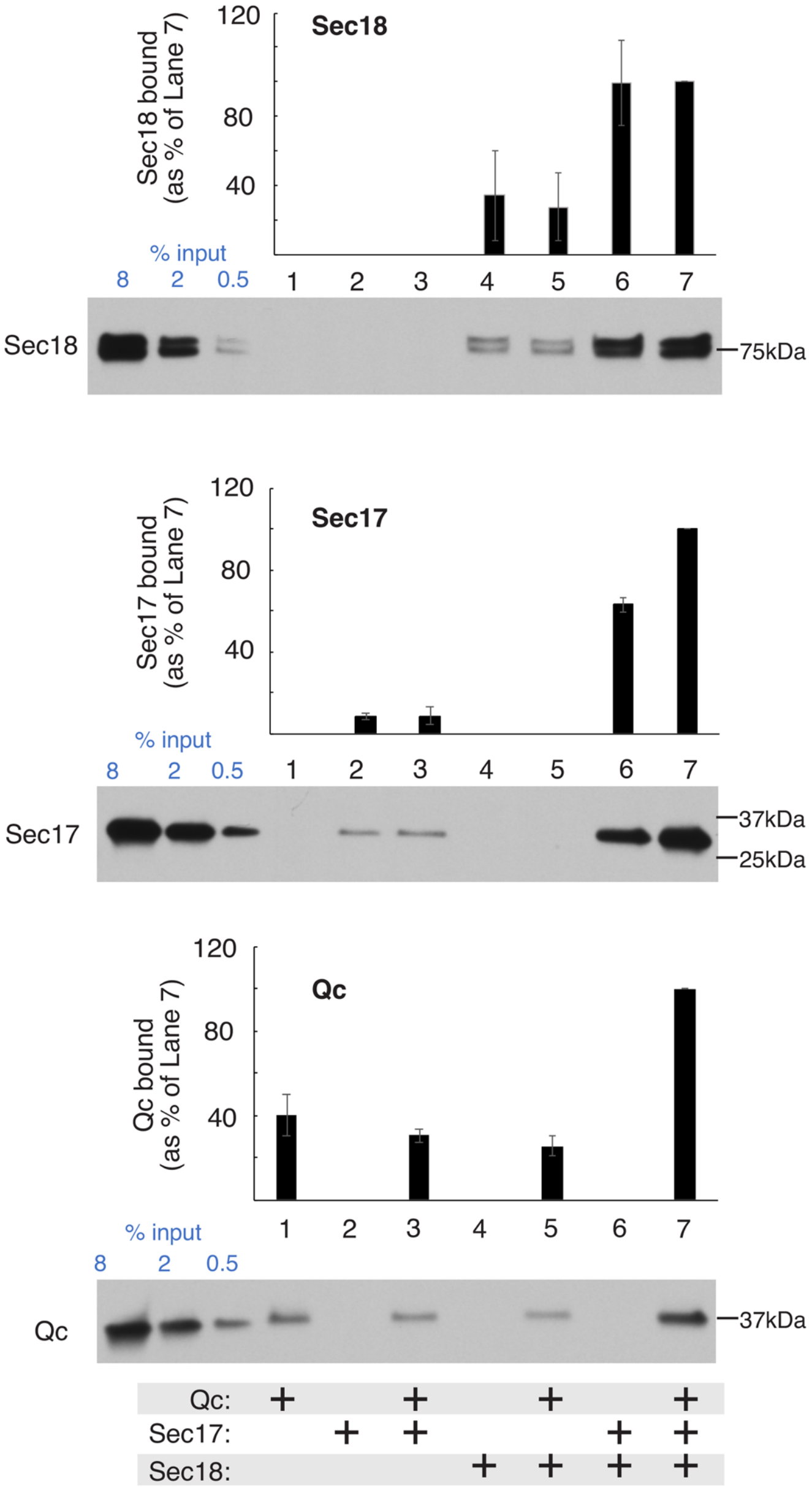

FIGURE 2: Binding cooperativity between Qc, Sec17, and Sec18. VML liposomes without any membrane proteins were prepared as described for binding assays. Assays were conducted as described with 1 mM Mg:ATPγS added to each reaction, and, where indicated, 1 µM Sec17, 250 nM Sec18, and 2 µM Qc, which had been buffer exchanged into 20 mM HEPES-NaOH pH 7.4, 500 mM NaCl, 10% glycerol, 1 mM DTT, 1 mM EDTA. Averages and standard deviations of triplicate experiments are shown.

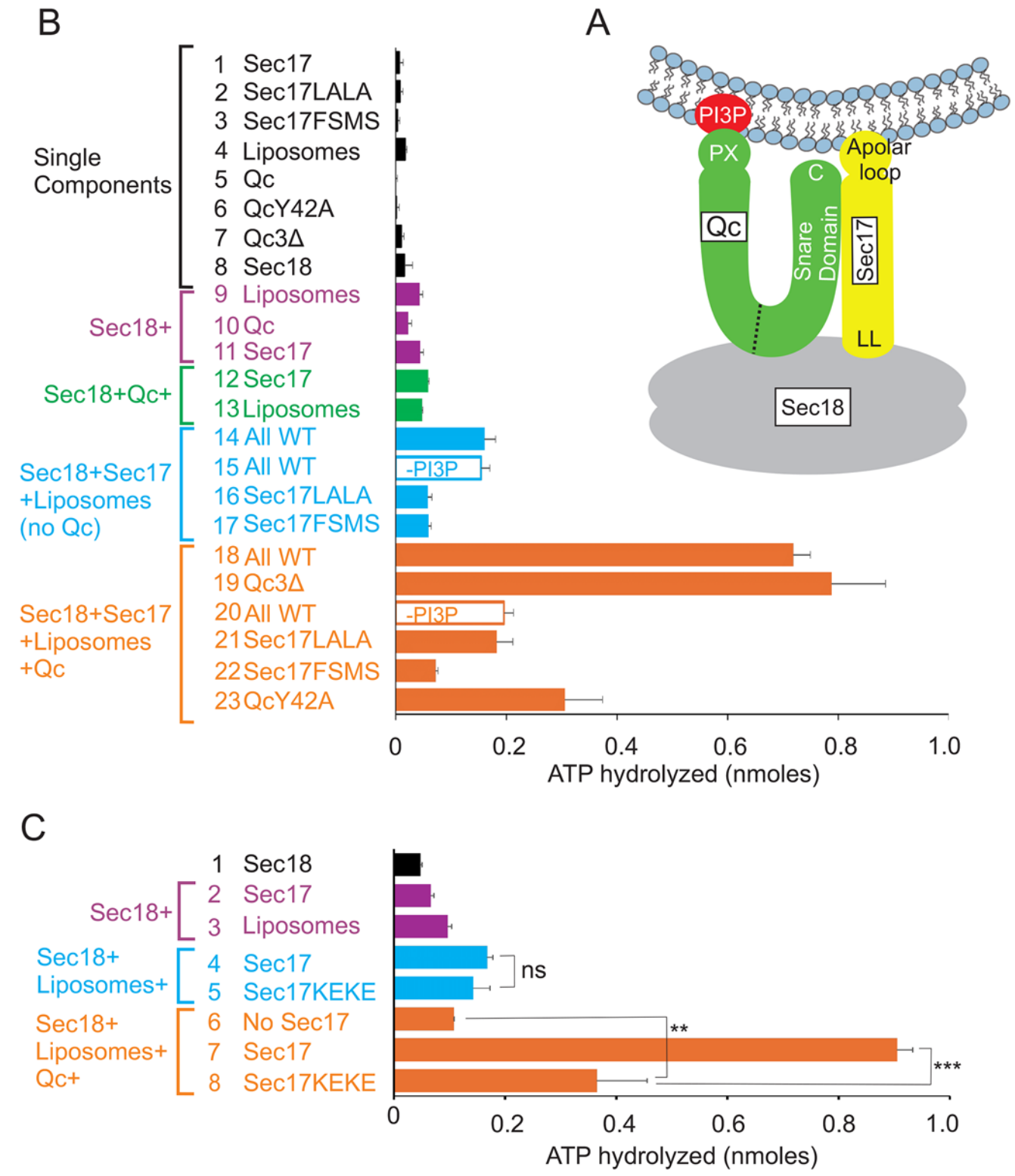

FIGURE 3: Assaying Sec18 associations by ATP hydrolysis. (A) Working model. Stimulation of Sec18 ATP hydrolysis by membrane-bound Sec17 and Qc requires specific interactions of Qc, Sec17, and Sec18 with each other and with membranes. (B) ATPase assays (20 µl) were performed for 30 min as described in *Materials and Methods* with 0.4 mM VML (vacuolar mixed lipid) liposomes, 50 nM Sec18, 1.5 µM Sec17 or its mutants, 1 µM Qc or its mutants, individually or in combinations as indicated. Averages and standard deviations of triplicate experiments are shown, with relevant statistics (Student *t* test) embedded in the text of *Results*. (C) Sec17 needs its SNARE-binding lysyl residues to stimulate Sec18 ATP hydrolysis when Qc is present. Assays of ATP hydrolysis, as described in *Materials and Methods*, were for 20 min and had (where indicated) 0.4 mM liposomes, 100 nM Sec18, the indicated concentrations of Sec17 or Sec17KEKE, and 1 µM Qc. In lane 7, with approximately 0.9 nmol ATP hydrolyzed, there were 0.03 nmoles Sec17 and 0.02 nmoles Qc, indicating turnover. Averages and standard deviations of triplicate experiments are shown, with relevant statistics (Student *t* test analysis).

Since Qc must bind membranes to stimulate the Sec18 ATPase, we compared the efficacy of soluble, wild-type Qc with Qc bearing a recombinant C-terminal transmembrane anchor (Xu and Wickner, 2012), termed Qc-tm, in assays of Sec18 ATPase activity with Sec17. ATP hydrolysis with Qc from 0 to 1000 nM (Figure 4A, lanes 6–10) with protein-free liposomes was compared with a reaction with liposomes bearing Qc-tm (lane 13). Incubations with equal stimulations of ATP hydrolysis by Sec18 (lanes 8 and 13) were analyzed by immunoblot and had the same concentrations of Qc (Figure 4A, top). Wild-type Qc, bound to membranes by its affinity for PI3P, has a similar stimulatory activity to transmembrane-anchored Qc.

To determine whether the only role of PI3P in support of Sec18 ATPase is to bind Qc to the membrane, we assayed Sec18 ATP hydrolysis in the presence or absence of Sec17 and either no liposomes (Figure 4B, black), liposomes without proteins (purple), proteoliposomes bearing Qc-tm (blue), or QcY42A-tm to ablate affinity for PI3P (orange). Liposomes without membrane proteins, with Qc-tm, or with QcY42A-tm were also prepared without PI3P. Without Sec17, proteoliposomes with Qc-tm only stimulated Sec18 to the same limited extent as liposomes without Qc-tm (Figure 4B, lanes 3 and 8 vs 1), but Qc-tm stimulated fusion in the presence of Sec17 (lanes 10 vs 5). PI3P is needed as the membrane receptor for wild-type Qc to stimulate Sec18 ATPase (lanes 6 vs 7), but is not needed with anchored Qc-tm (lanes 10 vs 11). Though the ablation of PI3P affinity through the Y42A mutation in Qc reduces its capacity to bind the membranes and stimulate Sec18 (Figure 3B, lanes 18 vs 23), the Y42A mutation has no effect when the Qc is

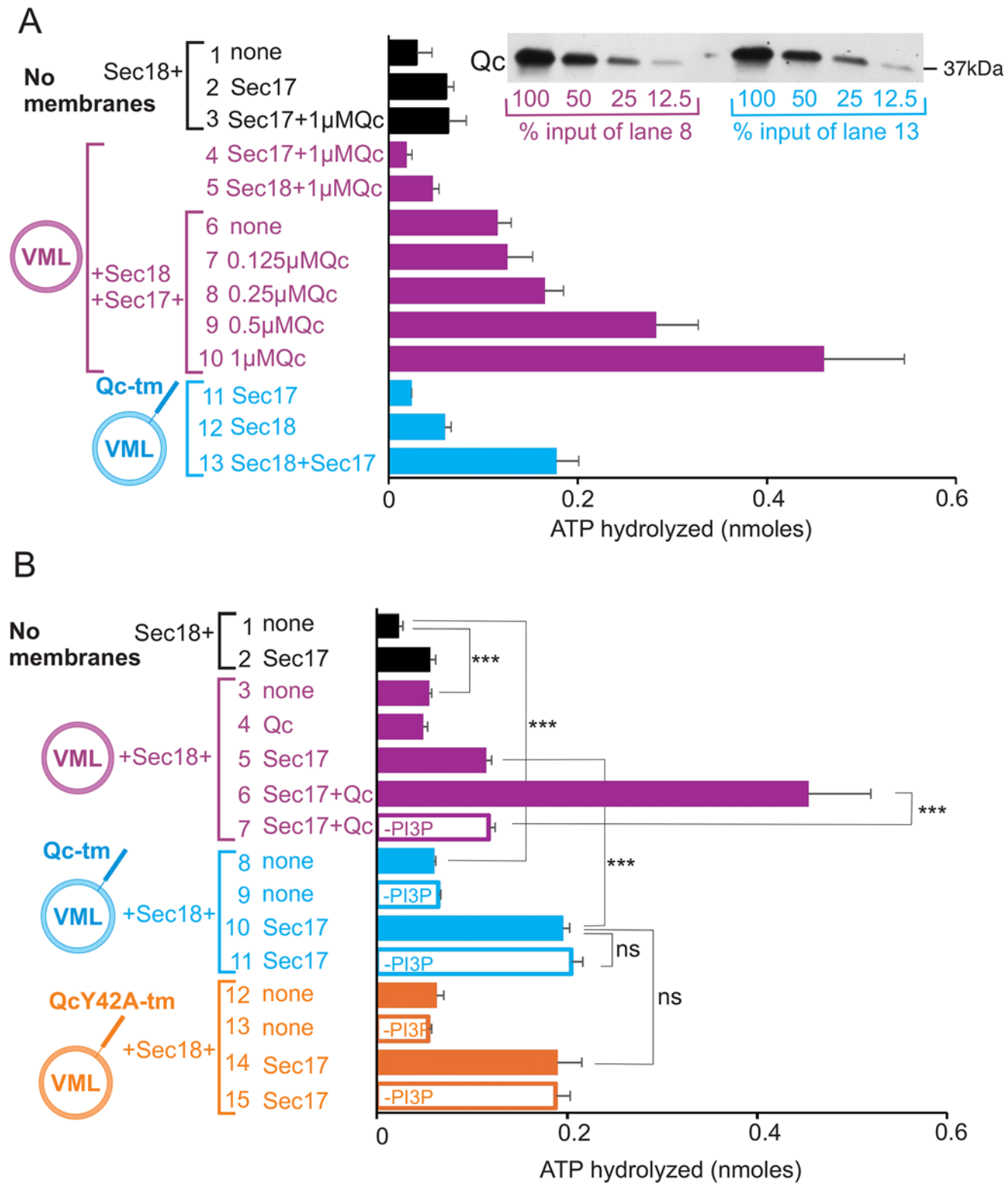

FIGURE 4: Stimulation of ATP hydrolysis by Qc-tm is as effective as Qc. (A) With the same amount of Qc in the incubation, there is equal Sec18 stimulation by Qc or Qc-tm. ATPase assays (10 min) were performed with 0.4 mM VML lipid, 1.5 μM Sec17, 50 nM Sec18, and 1, 0.5, 0.25, 0.125, or 0 μM Qc as indicated. Top, immunoblot; left four lanes are twofold dilutions of tube 8, the right four lanes are twofold dilutions of tube 13. (B) Anchored Qc-tm or QcY42A-tm does not need PI3P. ATPase assays (10 min, as described in *Materials and Methods*) had (where indicated) 0.4 mM membrane lipids (either complete vacuolar mixed lipids [VML] or with PI3P omitted), 1.5 μM Sec17, 50 nM Sec18, and 2 μM Qc. Averages and standard deviations of triplicate experiments are shown, with relevant statistics (Student *t* test analysis).

tm-anchored (Figure 4B, lanes 10 vs 14). The sole function of PI3P for stimulating Sec18 ATPase is therefore as a membrane binding site for Qc.

Which parts of the Qc-SNARE activate Sec18? Reconstituted proteoliposomes, termed RPLs, were prepared without protein, with Qc-tm, with Qc-tm deleted for its N-terminal PX domain and part of the remaining N-domain (Xu and Wickner, 2012; termed here QcΔPX-tm), or with Qc-tm deleted for its entire N-domain, leaving only the anchored SNARE domain (QcSD-tm). Each [proteo]liposome was incubated with Sec18. Without Sec17, there was little enhancement of the Sec18 ATPase by proteoliposomes bearing any of the three forms of Qc beyond that seen with protein-free liposomes (Figure 5A, lanes 5, 8, 11, 14). In the presence of Sec17, ATP hydrolysis was enhanced equally by Qc-tm or QcΔPX-tm (lanes 6 vs 9 or 12), while QcSD-tm (lane 15) gave maximal stimulation. Thus, the anchored SNARE domain of Qc interacts with Sec17 and Sec18 for maximal ATPase activity, modulated by the Qc N-domain.

Is the Qc SNARE domain uniquely active as a Sec17 ligand for activating Sec18 ATPase, or can other SNARE domains, singly or in combination, fulfill this function as efficiently? Proteoliposomes of vacuolar lipid composition were prepared without protein, with the R, Qa, Qb, or Qc-tm individual SNARE domains without their N-domains but with a C-terminal membrane anchor, or with several of these membrane-anchored SNARE domains. The low ATPase activity of Sec18 alone (Figure 5B, lane 2) is modestly enhanced by simply Sec17 (lane 3), less so by liposomes without Sec17, irrespective of their anchored SNARE(s) (lanes 2 vs 5, 8, 11, 14, or 17). Sec18 was further stimulated by the combination of Sec17 and liposomes (lane 6), with modest additional activity from anchored R- or Qa-SNARE domain (lanes 9 and 12). There was no additional stimulation by the anchored Qb SNARE domain (lane 15). The greatest stimulation of the Sec18 ATPase was by the anchored Qc SNARE domain (lane 18). Sec18 ATP hydrolysis stimulated by membrane-anchored Qc SNARE domain with Sec17 was not enhanced by the other anchored Q-SNARE domains (Figure 5C, lanes 15 vs 18), even with the anchored R-SNARE domain as well (Figure 5C, lanes 9 vs 18). Strikingly, the combination of anchored R, Qa, and Qb SNARE domains without Qc-tm gave less stimulation than Qc-tm alone (lanes 12 vs 18) and was barely above the signal with protein-free liposomes (lanes 12 vs 6) even though R, Qa, and Qb form a stable 3-SNARE complex (Song et al, 2020). Multiple anchored SNARE domains are not sufficient for full stimulation of Sec18 ATPase unless they contain the Qc-SNARE.

The same proteins and their interactions govern HOPS-dependent fusion and Sec18 ATP hydrolysis

To assay how Sec18, Sec17, Qc, and membranes with PI3P regulate fusion, proteoliposomes bearing Rab, R, and lumenal biotinylated phycoerythrin were mixed with those bearing Rab, Qa, and lumenal Cy5-streptavidin. Mixed soluble components were added: HOPS, soluble Qb (without a transmembrane anchor), Qc, Sec17, where indicated, Sec18, where indicated, and MgATP. While high concentrations of Sec17 can support fusion without Sec18 (Orr *et al.*, 2022), 100 nM Sec17 was added here to study its role in conjunction with Sec18. Fusion was assayed as the mixing of the proteoliposome lumenal compartments, allowing the two fluorescent proteins to bind and generate a strong FRET signal (Zucchi and Zick, 2011). With neither Sec17 nor Sec18, there is slow fusion, which is driven by complete SNARE zippering (Figure 6A, lane 1). There is minimal stimulation by 100 nM Sec17 (Figure 6A, lane 2). Mutant forms of Sec17 were used to selectively disable the Sec17 interactions with either the membrane, SNAREs, or Sec18. Fusion was statistically equivalent with either wild-type Sec17 (lane 2) or when the apolarity of the Sec17 N-domain loop was ablated by the FSMS mutation (lane 3), with Sec17LALA, which lacks the Sec17 binding site for Sec18 (lane 4), or when the Sec17 binding site for SNAREs was weakened by the KEKE mutation (lane 5). Without Sec17, there was little stimulation by Sec18 (lane 6 vs 1). The combination of Sec17 and Sec18 (Figure 6A, lane 7) gave a substantial stimulation of fusion (compare lane 7 with lanes 1, 2, and 6). This maximal fusion (lane 7) is inhibited by Sec17 mutants, which interfere with its interaction with membrane lipids (lane 8), with Sec18 (lane 9), or with SNAREs (lane 10). The inhibition from loss of Sec17 affinity for lipids or SNAREs is almost to the level of fusion seen without Sec17 at all (compare lanes 6 with 8 and 10). With Sec17LALA, which lacks affinity for Sec18, Sec18 does not affect fusion (compare lanes 4 and 9). Supplemental Figure S2, which contains examples of the kinetics of fusion (panels A and B), also shows that QcY42A, without affinity for membrane PI3P (Cheever *et al.*, 2001), does not support fusion at all, even with Sec17 or Sec18 (panel C). Thus, each protein (Sec17, Sec18, and Qc) and each interactive

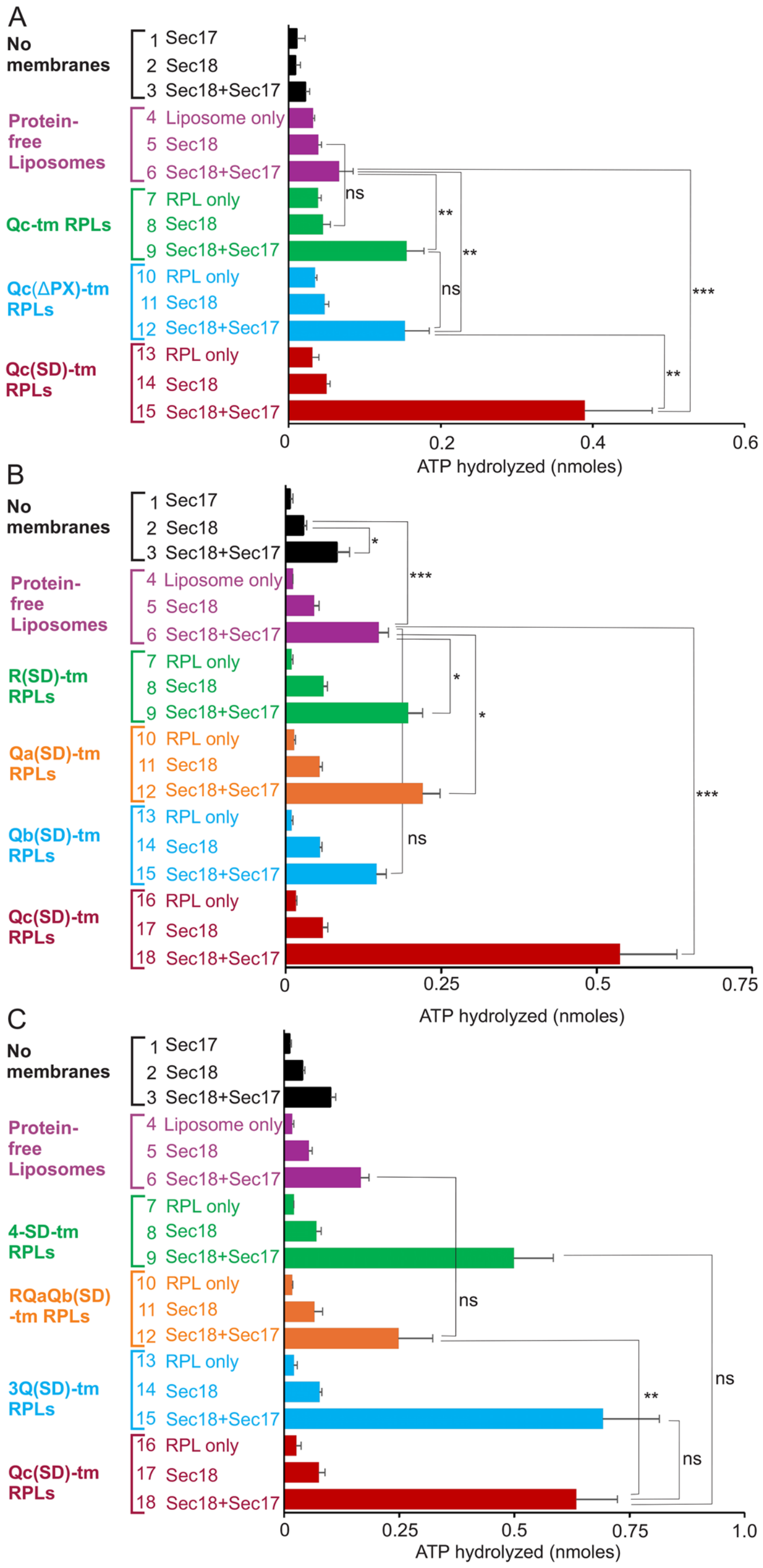

FIGURE 5: Potency of the Qc SNARE domain. (A) QcΔPX-tm or QcSD-tm are as effective as Qc-tm in supporting Sec17Sec18 ATPase. ATPase assays (10 min, as in *Materials and Methods*) had (where indicated) proteoliposomes with 0.4 mM lipids, 50 nM Sec18, and 500 nM Sec17. [Proteo]liposomes had no protein (purple) or a 1:2000 molar ratio to lipid of Qc-tm (green), QcΔPX-tm (blue), or QcSNAREDomain-tm (red) as indicated. (B) QcSD-tm is far more effective than R-, Qa-, or Qb- SD-tm. ATP hydrolysis assays (40 min, as in *Materials and Methods*) contained 3 μM Sec17, 50 nM Sec18, and no proteoliposomes (lane 3), protein-free liposomes (lane 6) or proteoliposomes bearing either R, Qa, Qb, or Qc SNARE Domain with a C-terminal transmembrane anchor (lanes 9, 12, 15, 18). As controls, lanes 5, 8, 11, 14, and 17 had the indicated proteoliposomes with 50 nM Sec18 but no Sec17, while lanes 4, 7, 10, 13, and 16 had only proteoliposomes without either Sec17 or Sec18. (C) QcSD-tm is more active than RQaQbSD-tm. ATPase assays (*Materials and Methods*, 40 min) had (where indicated) 50 nM Sec18, 3 μM Sec17, and proteoliposomes without membrane proteins (lanes 4–6), all 4 tm-anchored SNARE domains (7–9), and R, Qa, and Qb tm-anchored SNARE domains (10–12), all three tm-anchored Q SNARE domains (13–15), or QcSD-tm (16–18). Averages and standard deviations of triplicate experiments are shown, with relevant statistics (Student *t* test analysis).

domain needed for optimal ATP hydrolysis, as seen in Figure 3, also underlie optimal fusion.

ATP hydrolysis by Sec18 is not affected by HOPS

HOPS is essential for fusion, but does it regulate ATP hydrolysis? Fusion components were mixed and incubated under fusion assay conditions. EDTA was added to terminate ATP hydrolysis, and each sample was assayed for the inorganic phosphate released from ATP. Each separate reaction component only showed minimal phosphate contamination or generation (Figure 6B, lanes 1–5), but together there was substantial ATP hydrolysis (Figure 6B, lane 6). In assays where single reaction components were omitted, there was no effect of omitting HOPS (lane 9), but ATP hydrolysis was diminished by omission of Sec18, Sec17, or proteoliposomes (lane 6 vs 7, 8, 10). Since HOPS is required for *trans*-SNARE complex assembly, this confirms that HOPS, and presumably its receptor Rab and the *trans*-complex it assembles between R and Qa SNAREs, are not needed for the activation of Sec18 ATPase. The initial HOPS interactions with Rab and the R and Qa SNAREs are distinct from the initial interactions of Sec18, Sec17, Qc, and membranes.

DISCUSSION

Two layers of catalysis can enable Qc joining the SNARE complex. When there is no catalyst, that is, when Rab/R and Rab/QaQb proteoliposomes are tethered by a nonspecific agent such as either polyethylene glycol or dimeric glutathione-S-transferase fused to a lipid recognition domain such as PX or C1b, fusion has a very high EC50 for Qc and is extremely slow (Zick and Wickner, 2013; Song and Wickner, 2019). HOPS can directly bind Qc (Song *et al.*, 2020) and lower the EC50 of Qc for fusion (Zick and Wickner, 2013). When HOPS is limiting for fusion, Sec18 is also required to efficiently engage Qc (but not Qb) with low EC50 (Orr and Wickner, 2024). NSF/Sec18 has a direct binding site for Qc (White *et al.*, 2018). Qc:Sec18 association is enhanced by Sec17 through the Sec17 affinities for membranes (Zick *et al.*, 2015; Barnard *et al.*, 2017), for Qc (Figure 1) through the Qc SNARE domain (Figure 5), and for Sec18 (Söllner *et al.*, 1993). Fusion without HOPS is blocked by Sec17/Sec18, but Sec17 and Sec18 stimulate fusion with HOPS (Mima *et al.*, 2008). We propose that Qc is optimally recruited by two tandem steps, association with Sec17 and Sec18, followed by delivery to HOPS and the zippering-blocked complex of R and Qa. Zippering is thereby enabled, turning the apolar surfaces of the R and Qa SNAREs toward each other and away from their binding sites on the Vps33 subunit of HOPS (Baker *et al.*, 2015) to enable HOPS release.

Our studies are summarized in a working model (Figure 7). Membrane-bound Qc (Figure 7, part i) binds to the membrane-bound complex of Sec17 and Sec18 (ii) to form a complex of membrane-bound Sec18, Sec17, and Qc (iii). Independently, HOPS binds to the Rab on each membrane for tethering, then catalyzes the initial assembly of R and Qa on apposed membranes to form the HOPS-RQa *trans* complex, which binds Qb (iv). Without Qc, RQaQb zippering cannot proceed. The Sec18:Sec17:Qc complex

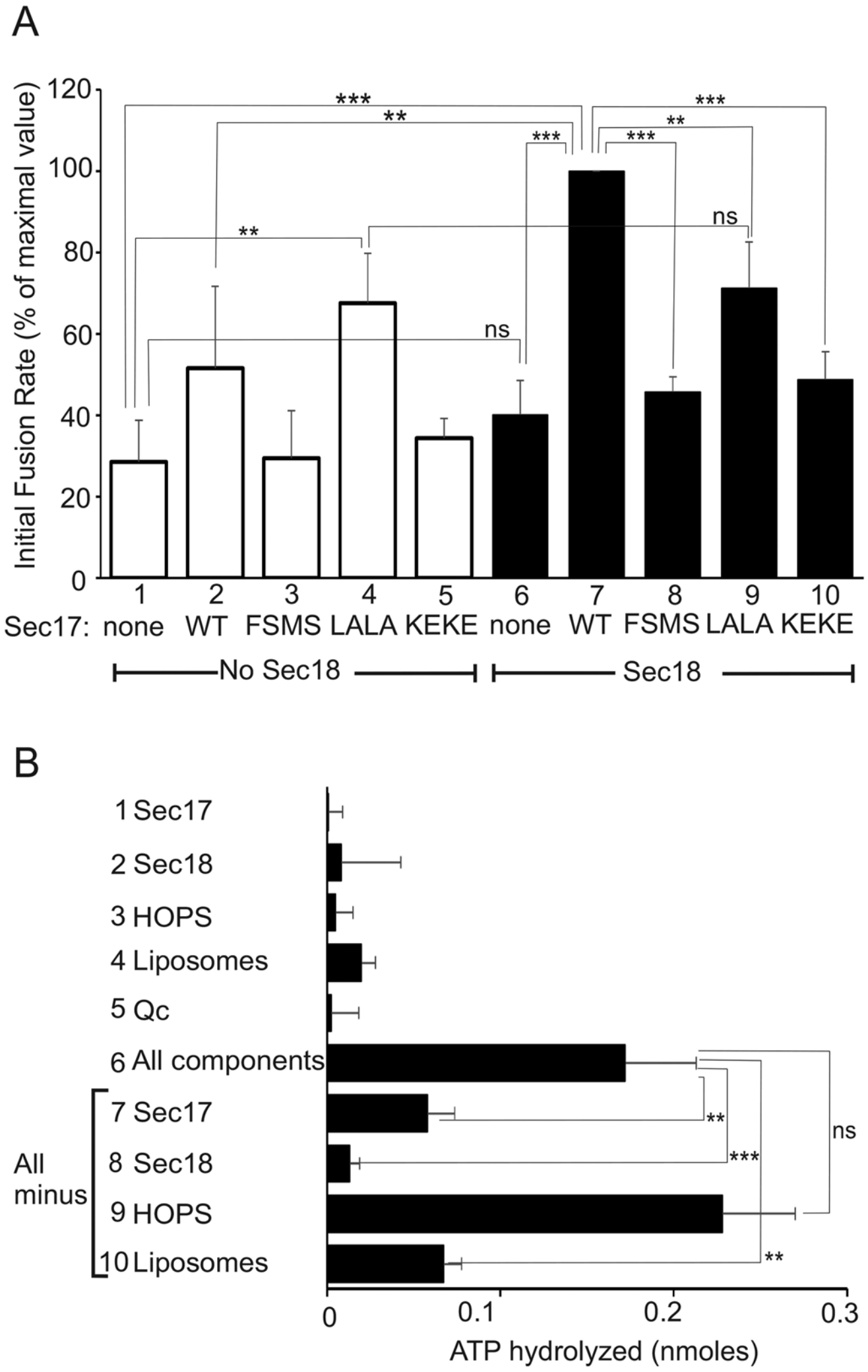

FIGURE 6: Sec17, Sec18, membranes, and their interactions affect membrane fusion as well as ATP hydrolysis. (A) Sec17 and Sec18 stimulate membrane fusion. Fusion was assayed (Orr and Wickner, 2024) as the FRET between biotin-phycoerythrin and Cy5-streptavidin, each initially entrapped in separate proteoliposome populations (Zucchi and Zick, 2011). Assays (20 µl) had proteoliposomes of vacuolar mixed lipid composition with Rab and R or with Rab and Qa (1:8000 molar ratio of Rab to lipids and 1:16,000 molar ratio of SNARE to lipid). Each assay was in the presence of excess nonfluorescent streptavidin to block any FRET signal from proteoliposome lysis. Mixed proteoliposomes were initially incubated with GTP and EDTA to strip bound guanine nucleotide, then given excess $MgCl_2$. Proteoliposomes and mixed soluble proteins (50 nM HOPS, 100 nM Sec17 where indicated, 50 nM Sec18 where indicated, 1 µM sQb, 20 nM Qc, and 1 mM MgATP) were preincubated in separate wells for 10 min at 27°C, then mixed with a multichannel pipettor. Modest concentrations of each Sec18 stimulant were employed to keep the highest levels of fusion responsive to each component. Assays were performed in 384 well plates (Item 4514, Corning, Kennebunk) in a Gemini XPS plate reader (Molecular Devices, Sunnyvale). Average and standard deviations of the initial rates are shown for triplicate experiments. Fusion was performed either without Sec18 (open bars) or with 50 nM Sec18 (filled bars) and either without Sec17 or with 100 nM Sec17, either wild-type, FSMS, LALA, or KEKE. (B) HOPS, the essential catalyst of tethering and *trans*-SNARE assembly for fusion, is not needed for ATP hydrolysis. Proteoliposomes were prepared for fusion assays as described in *Materials and Methods* , but each internal fluorescent protein stock had first been dialyzed twice against a 200-fold volume excess of Rb150 to remove phosphate. Fusion incubations, as described above, had Rab/R and Rab/QaQb proteoliposomes (333 µM lipids, 1:16,000 molar ratios of SNAREs:lipids, 1:8000 of Rab:lipids) and 100 nM HOPS, 300 nM Sec17, 50 nM Sec18, 1 mM MgATP, and 1 µM Qc except where omitted as noted. Incubations (20 min, 23°C, 0.5 ml conical plastic tube) were followed by assay of inorganic phosphate as described in *Materials and Methods*. Averages and standard deviations of triplicate experiments are shown, with relevant statistics (Student *t* test analysis).

binds the HOPS:R:QaQb complex by some combination of the affinities of Qc for the other SNAREs and for HOPS and of Sec17 for HOPS and for the entire SNARE complex. HOPS is displaced during this assembly, yielding the Sec18:Sec17:*trans*-4-SNARE prefusion complex (v). Slow fusion can be supported by the zippering of the *trans*-SNARE complex without Sec17 or Sec18 (Fukuda *et al.*, 2000; Mima *et al.*, 2008; Song *et al.*, 2017) or by Sec17 and Sec18 without completion of zippering (Schwartz and Merz, 2009; Zick *et al.*, 2015; Song *et al.*, 2021), but rapid fusion from this prefusion complex (v) needs both. Although the stoichiometry and structures of each of these complexes are not known, each is supported by prior and current studies.

Membrane-bound Qc (i): The Qc-SNARE binds membranes by the affinity of its N-terminal PX domain for PI3P (Cheever *et al.*, 2001). Qc is comprised of an N-terminal PX domain, a helical coil region, a hinge region, and a C-terminal SNARE domain. The affinity of the Qc PX domain for PI3P (Cheever *et al.*, 2001) is needed for wild-type Qc binding to membranes and optimal activation of Sec18 (Figure 3B), but PI3P is not needed for Sec18 stimulation with Qc-tm, which is integrally anchored (Figure 4). The PX domain is likely folded back on the SNARE domain to bind the membrane adjacent to the C-terminus of Qc and other Q-SNAREs. Structure predictions from alpha-fold suggest that Qc has a central disordered region compatible with this model, but this remains to be tested directly. To support fusion, each SNARE domain must remain oriented with its C-terminus near the membrane to engage Sec17 and the other 3 SNAREs, as shown in the 20s structure (Zhou *et al.*, 2015).

Membrane-bound complex of Sec17 and Sec18 (ii): Independent of Qc, Sec18 and several Sec17 assemble and undergo interdependent binding to membranes (Orr and Wickner, 2022) by the product of the affinity of the apolar loop of each of the several Sec18-bound Sec17 molecules for lipid (ibid). Membrane-bound Sec18:3Sec17 stimulates fusion at low Sec17 levels and eliminates the cooperativity of fusion for Sec17 concentration (ibid). Lipid and Sec17/SNAP can cooperate to enhance 4-SNARE complex disassembly by Sec18/NSF (Winter *et al.*, 2009), and they even stimulate Sec18 ATP hydrolysis without SNAREs (Figure 4A, lane 1 vs 6), highlighting the sensitivity of Sec18 to each of its ligands.

Sec17:Sec18:Qc complex on membranes (iii): Qc has direct physical and functional interactions with Qa and Qb in the 3Q or 4-SNARE bundle (Sutton *et al.*, 1998), with HOPS (Song et al., 2020), PI3P (Cheever *et al.*, 2001), Sec17 (Figure 1A), and Sec18 (White *et al.*, 2018). Qc association with the 3Sec17:Sec18 complex (Figure 2A) to form Qc:3Sec17:Sec18 may exploit the high affinity of Sec17 for Qc (Figure 1A). More Qc binds to membranes with Sec17 and Sec18 (Figure 2, lane 7) than with either Sec17 or Sec18 alone (Figure 2, lanes 3 and 5). The anchored Qc SNARE

domain in Qc:3Sec17:Sec18 activates the Sec18 ATPase (Figure 5A, lane 15). The C-terminal third of this SNARE domain is not needed for Sec18 activation (Figure 3B, lane 19), in accord with only the N-terminal portion of the synaptic SNARE complex being engaged with NSF (White *et al.*, 2018). Optimal Qc:Sec17 interaction on membranes may rely on Qc being in a loop-like conformation with the C-terminus of its SNARE domain adjacent to its N-terminal PX binding site for PI3P. In this conformation, the SNARE domain of membrane-bound Qc can bind Sec17 and be properly presented to Sec18. The affinity of the Qc PX domain for PI3P (Cheever *et al.*, 2001) is needed for wild-type Qc binding to membranes and activation of Sec18. Qc needs its PI3P recognition to support fusion (Supplemental Figure S2, Panel C), but PI3P is not needed for Sec18 ATPase stimulation with Qc-tm, which is integrally anchored (Figure 5A). Three recognition surfaces on Sec17 are critical, both for Sec17 stimulation of fusion in the presence of Sec18 and for its activation of the Sec18 ATPase. Both activities are diminished by either the Sec17FSMS abrogation of N-loop apolarity (Figures 3B and 6A), by diminished SNARE affinity of Sec17KEKE (Figures 3C and 6A), and by diminished Sec18 affinity of Sec17LALA (Figures 3B and 6A).

HOPS:RQaQb complex (iv): The HOPS:RQaQb *trans*-complex between tethered membranes has been studied and reconstituted with proteoliposomes, supporting strikingly rapid fusion upon engaging Qc (Song *et al.*, 2020).

Assembly of the prefusion complex (v): 3Sec17:Sec18:Qc association with the HOPS:trans-RQaQb complex may exploit HOPS recognitions of Qc (Song *et al.*, 2020; Zick and Wickner, 2013), Sec17 (Orr and Wickner, 2022), Sec18 (Orr and Wickner, 2024), Qc engagement with the other SNAREs, and Sec17 affinity for a complex of RQaQb (Figure 1, lane 9). As Qc enters and completes the *trans*-SNARE complex to allow zippering, both SNARE zippering and Sec18 and Sec17 may displace HOPS (Collins *et al.*, 2005; Schwartz *et al.*, 2017). The SNAREs on isolated vacuoles are in complex with either HOPS or with Sec17 but not with both (Collins *et al.*, 2005), suggesting that HOPS displacement by Sec17:Sec18:Qc is efficient. Since Sec18 dramatically lowers the EC50 of Qc for fusion (Orr and Wickner, 2024), Qc either associates with Sec17:Sec18 as a prelude to joining the other SNAREs (Figure 7) or Sec17:Sec18 joins the HOPS:RQaQb complex and enhances its affinity for Qc. Further studies are needed to test these alternative models. It is not known whether membrane fusion at other organelles relies on Sec17 and Sec18 for efficient delivery of their Qc to the other SNAREs.

MATERIALS AND METHODS

Lipids were from Avanti Polar Lipids (Alabaster, AL), Echelon Biosciences (Salt Lake City, UT), Sigma-Aldrich (Burlington, MA), and Invitrogen (Eugene, OR). Biotinylated R-phycoerythrin and glycerol were from Invitrogen, and Cy5-streptavidin from SeraCare (Milton, MA). Dialysis tubing was from Repligen (Waltham, MA) and BioBeads from Bio-Rad (Hercules,CA). n-Octyl-β-D-glucopyranoside (ß-octylglucoside) was from Anatrace (Maumee, OH). HEPES, Thesit, Histodenz, ATP, ATPγS, IPTG, and defatted bovine serum albumin (BSA) were from Sigma-Aldrich. Dithiothreitol (DTT) was from Research Products (Mt. Prospect, IL).

Proteins

GST-Vam3, Vti1, and Nyv1 were purified as described (Mima *et al.*, 2008). Vti1 and Nyv1 were buffer exchanged into Rb150 (20 mM HEPES-NaOH, pH 7.4, 150 mM NaCl, 10% glycerol) + 1% ß-octylglucoside (Zucchi and Zick, 2011).

The Qc-SNARE Vam7 (Schwartz and Merz, 2009) was purified as described (Starai *et al.*, 2007) with modifications. The plasmid encoding Qc (Schwartz and Merz, 2009) was used as template DNA to make the QcY42A point mutant using the primers from Fratti *et al.* (2007). For the wild-type Qc and the Y42A mutant, plasmid DNA was transformed into C43(DE3)-RP cells and plated on LB + kanamycin and chloramphenicol. A clone was grown in 200 ml LB (L Broth) + Kan/Cam overnight with shaking at 37°C. In the morning, 60 ml was transferred into each of three 4-l baffled flasks containing 2 l of Terrific Broth, TB salts, Kan, and Cam, and shaken at 37°C to an OD of 2.0. Expression was induced with 1 mM IPTG, and cultures were grown for 4 h with shaking. Cells were sedimented (JLA10.5 rotor, 5,000 rpm, 5 min, 23°C), then resuspended in 80 ml of Buffer A (20 mM HEPES-NaOH, pH 8.0, 0.5 M NaCl, 0.1% Triton-X100, 1 mM EDTA) and French pressed twice. Lysates were centrifuged in a Beckman 60Ti fixed angle rotor (50,000 rpm, 30 min, 2°C). Supernatants were divided between two Falcon tubes, each with 14 ml chitin resin (NEB, Ipswich, MA) pre-equilibrated in Buffer A, nutated for 1 h at 4°C, then poured into a 2.5 cm diameter column. After the resin settled and the solution drained, it was washed with 100 ml wash buffer (Buffer A minus the Triton). Cleavage buffer (40 ml, Buffer A without Triton and with 50 mM DTT) was run through the resin at 4°C, then the column was plugged and moved to room temperature overnight. In the morning, 6 ml fractions were collected, eluting with 100 ml of wash buffer, put on ice, assayed for protein, and the protein peak was pooled. The eluted Qc was aliquoted into PCR strips, snap frozen, and stored at −80 °C. The N-terminal His_6 tag was removed by TEV cleavage during the proteoliposome preparation process. Qc3Δ (Schwartz and Merz, 2009) was purified as above, except that the expression strain was Rosetta2(DE3), growth and IPTG induction were at 30°C, and Buffer A was replaced by Buffer B, composed of 20 mM TrisCl, pH 8.0, 0.5M NaCl, 10% glycerol, and 1 mM EDTA. PMSF was added to 1 mM to the cell lysate. The chitin resin was nutated at 4°C for 3 h, poured into the column, and the solution drained. The resin was washed with 30 ml of Buffer B, then 60 ml of Buffer B with 1.5M NaCl, then 60 ml of Buffer B, all at 4°C. Cleavage buffer was Buffer B + 50 mM DTT.

Plasmids expressing Sec17, Sec17LALA (Schwartz and Merz, 2009), Sec17KEKE and Sec17FSMS (Schwartz *et al.*, 2017) were transformed into Rosetta2(DE3) and plated on LB+Ampicillin. A clone was grown overnight in 400 ml LB+Amp in a 2L flask with shaking at 37°C. In the morning, 125 ml portions were transferred into three 4 l flasks which each had 2 l of Terrific Broth with TB Salts and Amp. Cultures were grown to OD_{600} of 1.5, then shifted to an 18°C shaker where they received 0.5 mM IPTG and were shaken overnight. Cells were collected by centrifugation (JLA10.5 rotor, 5000 rpm, 5 min, 3°C) and resuspended in 120 ml Buffer C (20 mM TrisCl, pH 8.0, 0.5 M KCl, 1 mM EDTA). PMSF (200 μM) and 0.01 volume of 100x PIC (100x protease inhibitor cocktail: 46 μg/ml leupeptin, 350 μg/ml pepstatin A, 240 μg/ml pefablock SC in dimethylsulfoxide) were added, and the cells were lysed by French press. The lysate was centrifuged (Beckman 60Ti rotor, 55,000 rpm, 30 min, 2°C). The supernatant was nutated with 60 ml of chitin resin previously equilibrated with Buffer C for 2 h at 4°C, then poured into a 5 cm diameter column, drained, and washed with 500 ml Buffer C. Cleavage buffer (Buffer C + 40 mM DTT) was passed through the column (100 ml) and the column was plugged and moved to room temperature overnight. The next morning, 125 ml of Buffer C was added, and 10 ml fractions were collected. The pooled protein fractions were concentrated in a YM-10 Centriprep,

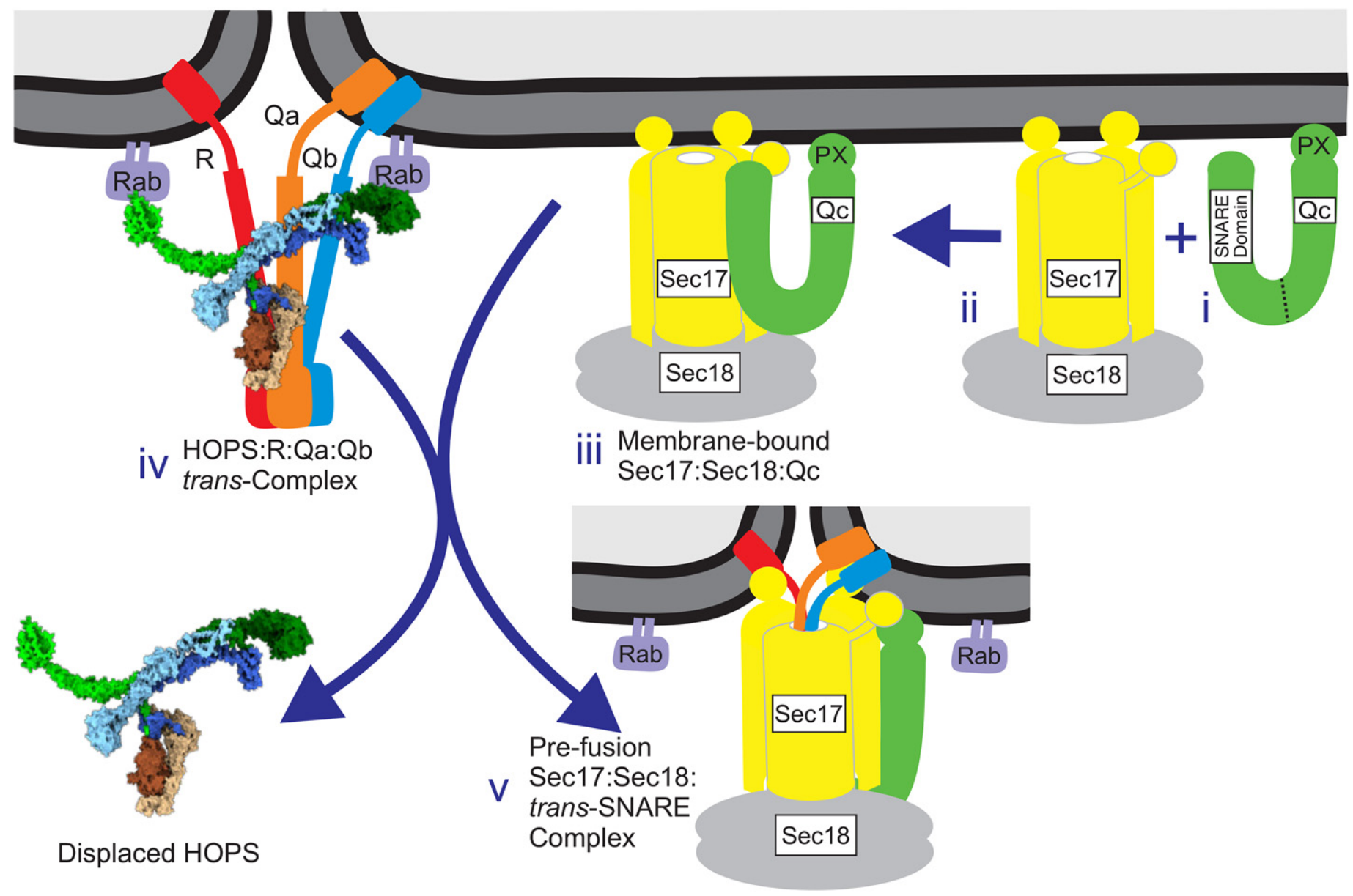

FIGURE 7: Current working model of the Sec17:Sec18:Qc complex and the HOPS:R:Qa:Qb complex combining while displacing HOPS, forming the prefusion complex. There is evidence in support of each intermediate: (i) Qc is membrane-bound by the affinity of its N-terminal PX domain for PI3P (Cheever *et al.*, 2001). (ii) Sec18 and Sec17 assemble into a 3Sec17:Sec18 complex which binds to lipid by the Sec17 apolar loops (Orr and Wickner, 2022), thereby activating the Sec18 ATPase (Figure 3). (iii) Qc binds to Sec17:Sec18 at the membrane (Figure 2) by its affinity for Sec17 (Figure 1A) and for Sec18/NSF (White *et al.*, 2018), further activating ATP hydrolysis (Figure 3). (iv) HOPS catalyzes the assembly of R:QaQb rapid-fusion intermediates (Song *et al.*, 2020). (v) HOPS and Sec17 never associate with the same SNARE complexes (Collins *et al.*, 2005), and Sec17 can displace HOPS from SNAREs (Collins *et al.*, 2005; Schwartz *et al.*, 2017).

then dialyzed overnight into Rb150, aliquots frozen in liquid nitrogen, and stored at −80°C.

His_6-Sec18 (Haas and Wickner, 1996) was purified as follows. XL1 Blue/ pQE9-His_6-Sec18 cells were grown in 300 ml LB + Ampicillin and Tetracycline at 37°C overnight with shaking. In the morning, three 90 ml portions were each added to 3 l of LB with Amp and Tet in 6 l flasks and grown to an OD_{600} of 0.8. IPTG was added to 0.5 mM, and growth continued for 4 h to an OD 1.25. Cells were collected (JLA10.5 rotor, 5000 rpm, 5 min) and resuspended in 300 ml lysis buffer (100 mM HEPES-KOH, pH 7.0, 500 mM KCl, 5 mM ATP, 5 mM $MgCl_2$, 0.014% ß-mercaptoethanol, 1x PIC) with 0.5 mM PMSF. Washed cells were sedimented a second time, then resuspended in 150 ml of the same lysis buffer with PMSF, then French pressed twice and centrifuged (Beckman 60Ti, 50,000 rpm, 1 h, 2°C). The cytosol was mixed with 12 ml of Qiagen Nickel-NTA resin in Buffer D (20 mM HEPES-KOH, pH 7.0, 480 mM KCl, 0.5 mM ATP, 10% glycerol, 1 mM $MgCl_2$, 0.014% ß-mercaptoethanol, 20 mM imidazoleCl), nutated for 1 h at 4°C, then poured into a 1.5 cm column, drained, and washed with 100 ml Buffer D. The protein was eluted with 50 ml of Buffer E (20 mM HEPES-KOH pH 7.0, 0.5 mM ATP, 10% glycerol, 1 mM $MgCl_2$, 0.014% ß-mercaptoethanol, 500 mM imidazoleCl) and 2 ml fractions were collected. The pooled protein was frozen in liquid nitrogen in 1 ml Eppendorf tubes and stored at −80°C for a later gel filtration step, as follows: Five 1 ml aliquots of the purified His_6-Sec18 were thawed and centrifuged for 5 min at 4°C in a tabletop microfuge. They were pooled and applied to a 2.5 × 40 cm column with 196 ml of Superdex 200 prep grade resin (GE Healthcare, Uppsala, Sweden) in 20 mM PIPES-KOH, pH 6.8, 0.2 M sorbitol, 125 mM KCl, 5 mM $MgCl_2$, 2 mM Na_2ATP, 2 mM DTT, 10% glycerol at 4°C and 3 ml fractions were collected. The gel filtered protein was pooled and snap frozen in PCR strips and stored at −80°C.

DNA encoding the Qa, Qb, Qc, and R SNARE domains and transmembrane anchors, optimized for *E. coli* expression, was synthesized and cloned by Genewiz-Azenta (South Plainfield, NJ). All fragments were cloned into a pMBP-Parallel1 vector using the BamHI and SalI sites. The QaSNAREDomain-tm (QaSD-tm) includes both the 18 amino acids before the SNARE Domain of Vam3, which have been shown to be important for fusion (Song and Wickner, 2017), and its original transmembrane domain, amino acids 181-283. The construct QbSNAREDomain-tm corresponds to the amino acids 133-217 of Vti1, including the SNARE domain and its original transmembrane domain. The construct QcSNAREDomain-tmQb includes the amino acids 259-316 of Vam7 followed by the transmembrane domain of Vti1, amino acids 195-217. The construct RSNAREDomain-tm includes the SNARE domain and the original transmembrane domain of Nyv1, amino acids 165-253. For QcY42A-tm, DNA encoding the Qc SNARE containing the changed codon for amino acid 42 (Tyrosine to Alanine) and the transmembrane domain of the Qb SNARE was synthesized and cloned by Genewiz-Azenta. The fragment was codon optimized for expression in *E. coli* and cloned into the pMBP-Parallel1 vector using the BamHI and SalI sites. The final construct contains amino acids 1-316 of Vam7 followed by the transmembrane domain of Vti1, amino acids 195-217. All anchored SNARE domain proteins were expressed in BL21 STAR cells (Invitrogen, Waltham, MA) in the presence of 100 µg/ml Ampicillin. The anchored QcY42A protein was expressed in Rosetta2(DE3) pLysS cells (Millipore Sigma, Burlington, MA) in the presence of 100 µg/ml Ampicillin and 35 µg/ml Chloramphenicol. The purification steps

were identical for the anchored SNARE domains and QcY42A. Cells containing the MBP-tagged proteins were grown overnight in 100 ml of LB medium with antibiotics at 37°C and shaking. In the morning, the 100 ml cell growth was poured into 3 l of fresh medium plus antibiotics. Cells were grown to OD_{600} of 1, IPTG was added to 1 mM, and incubation with shaking continued for 5 h. Cells were harvested by centrifugation (JLA10.5 rotor, 5000 rpm, 5 min) and resuspended in 30 ml of 20 mM TrisCl, pH 8, 200 mM NaCl, 1 mM EDTA, 1 mM DTT, 0.5 mM PMSF, and 1X PIC. Cell suspensions were French pressed twice and centrifuged in a Beckman 60Ti rotor (50,000 rpm, 45 min, 4°C). The membrane fraction, containing the anchored proteins, was resuspended in 23 ml of 20 mM TrisCl, pH 8, 200 mM NaCl, 1 mM EDTA, 1 mM DTT, 0.5 mM PMSF, 1X PIC and 1% Triton X100, nutated for 1 h at 4°C to solubilize the membranes and centrifugated again (50,000 rpm, 1 h, 4°C). The supernatant was added to 15 ml of amylose resin equilibrated with 20 mM TrisCl, pH 8, 200 mM NaCl, 1 mM EDTA, 1 mM DTT, and 1% Triton X100, nutated for 2 h at 4°C, and poured into a column. After the resin had settled and the column drained, the resin was washed with 100 ml of the same buffer used for equilibration, and protein was eluted with 40 ml of 20 mM HEPES-NaOH, pH 7.4, 200 mM NaCl, 10% glycerol, 1 mM DTT, 1% ß-octylglucoside, and 10 mM maltose. The eluate was collected in 2 ml fractions, and protein concentrations were determined by Bradford. Small aliquots of proteins were frozen in liquid nitrogen and stored at −80°C.

MBP-Qc-tm and the QcΔPX-tm variant (Xu and Wickner, 2012) were purified as described. HOPS and the soluble Vti1 (Zick and Wickner 2013), soluble Vam3 and Ypt7-tm (Song *et al.*, 2020), and soluble GST-Nyv1 (Thorngren *et al.*, 2004, Song *et al.*, 2020) were purified as described.

Systematic names and ID numbers of all proteins used in this work according to Saccharomyces Genome Database (Saccharomyces Genome Database | SGD): Qa SNARE or Vam3 (YOR106W, SGD:S000005632), Qb SNARE or Vti1 (YMR197C, SGD:S000004810), Qc SNARE or Vam7 (YGL212W, SGD: S000003180), R SNARE or Nyv1 (YLR093C, SGD:S000004083), Rab or Ypt7 (YML001W, SGD:S000004460), Sec17 (YBL050W, SGD:S000000146), Sec18 (YBR080C, SGD:S000000284). HOPS subunits: Vps11 or Pep5 (YMR231W, SGD:S000004844), Vps16 or Vam9 (YPL045W, SGD:S000005966), Vps18 or Pep3 (YLR148W, SGD:S000004138), Vps33 or Pep14 (YLR396C, SGD:S000004388), Vps39 or Vam6 (YDL077C, SGD:S000002235), Vps41 or Vam2 (YDR080W, SGD:S000002487).

Proteoliposome preparation

Proteoliposomes were prepared as in Zick and Wickner (2016) with modifications. For each 1 ml preparation of liposomes, 4 mM vacuolar mimic lipids in chloroform and 50 mM ß-octylglucoside were mixed in a glass vial. Vacuolar mimic lipid contains 47.6% 1,2-dilinoleoyl-*sn*-glycero-3-phosphocholine (Avanti 18:2 PC item 850385C), 18% 1,2-dilinoleoyl-*sn*-glycero-3-phosphoethanolamine (Avanti 18:2 PE item 850755C), 18% L-α-phosphatidylinositol (Avanti soy PI item 840044C), 4.4% 1,2-dilinoleoyl-*sn*-glycero-3-phospho-L-serine (Avanti 18:2 PS item 840040C), 2% 1,2-dilinoleoyl-*sn*-glycero-3-phosphate (Avanti 18:2 PA, item 840885C), 8% ergosterol (Fluka Sigma item 45480-10G-F), 1% 1,2-dipalmitoyl-*sn*-glycerol (Avanti 16:0 DG item 800816C), and 1% Di-*myo*-phosphatidylinositol 3-phosphate (Echelon PI(3)P, diC16, item P-3016, dissolved in 1:2:0.8 chloroform:methanol:water). Lipid detergent mixtures were dried under a stream of nitrogen for 30 min. Solvent was further removed by centrifugation under vacuum for 3 h. Lipid pellets were resuspended by nutation at room temperature in 400 µl of 50 mM HEPES-NaOH, pH 7.4, 375 mM NaCl, 25% glycerol, 2.5 mM $MgCl_2$.

To prepare proteoliposomes for fusion assays, SNAREs were added to the 400 µl of reconstituted lipids at a 1:16,000 SNARE to lipid molar ratio, and the Rab Ypt7-tm was at a 1:8000 molar ratio to lipid. His_6-TEV (1 µM) was added to each preparation to remove purification tags. Suspensions with the R SNARE also received 250 µl of 16 µM R-phycoerythrin solution (Invitrogen item P811), while suspensions with any Q SNARE received 250 µl of 32 µM Cy5-streptavidin solution (SeraCare item 5270-0023). Water was added to bring the volume of each preparation to 1 ml, but it was mixed with the lipid-detergent solution before proteins or fluorescent probes were added. To prepare liposomes for assays of ATP hydrolysis or lipid binding, the fluorescent probes were omitted, their volumes replaced by water, and the SNAREs, when present, were added at a 1:2000 protein to lipid ratio. For lipid binding assays, 1% of the 18:2 PC was replaced by 1% Rhodamine-DHPE (Invitrogen item L1392). The lipid-detergent-protein solutions were nutated for 30 min, 4°C, then dialyzed overnight, in the dark, at 4°C in 12 mm (flat width) 25kD MWCO dialysis tubing (Repligen item 132550), against 200 ml (per liposome prep) of Rb150+Mg (20 mM HEPES-NaOH, pH 7.4, 150 mM NaCl, 10% glycerol, 1 mM $MgCl_2$) with 1 g BioBeads SM-2 (BioRad item 152-3920) per 200 ml dialysis buffer. Liposomes were harvested into 2 ml Eppendorf tubes on ice, mixed with an equal volume of 70% Histodenz in modified Rb150+Mg (containing only 2% glycerol), and transferred to Beckman 11 × 60 mm ultraclear centrifuge tubes. These were overlaid with 25% Histodenz in modified Rb150+Mg to the 3.8 ml mark on the tube. A final layer of 600 µl Rb150+Mg was added to the top. Tubes were centrifuged (60Ti, 55,000 rpm, 90 min, 4°C). Liposomes were harvested onto ice and lipid phosphorus concentration determined (Chen *et al.*, 1956). Liposomes were diluted with Rb150+Mg to 2 mM lipid phosphate, aliquoted (30 µl) into PCR strips, and frozen and stored in liquid nitrogen.

Binding assays

Assays of proteins bound to liposomes were conducted as described (Orr and Wickner, 2022). Liposomes were prepared with VML lipids and 1% Rhodamine-DHPE as described above. In brief, 30 µl reactions in Rb150 contained 0.5 mM lipid, 0.04% defatted BSA, 1 mM $MgCl_2$, and soluble proteins as indicated. These were incubated in PCR strips in a 27°C water bath for 1 h. Reactions received 90 µl of 54% Histodenz in modified Rb150+Mg with only 2% glycerol and were gently vortexed. A portion (80 µl) of each was transferred to 7 × 20 mm ultracentrifuge tubes, over-layered with 80 µl of 35%, then 80 µl of 30% Histodenz in modified Rb150+Mg, then 50 µl of Rb150+Mg. The gradients were centrifuged in a Beckman TLS55 swinging bucket rotor (55,000 rpm, 30 min, 4°C). The top 80 µl was harvested, solubilized by adding Thesit to 0.125%, nutated for 30 min, and the percentage of recovery was determined by Rhodamine fluorescence, as compared with the likewise solubilized starting samples. Samples were boiled in SDS–PAGE sample buffer with ß-mercaptoethanol and analyzed by Western blot. Assays were performed in triplicate, and UN-SCAN-IT software (Silk Scientific, Orem, UT) was used to quantify bands. Averages and standard deviations are presented.

ATP hydrolysis assays

ATP hydrolysis was assayed with a phosphate assay kit ab270004 from Abcam (Cambridge, UK) according to the manufacturer's instructions. Before the assay, Sec18 was freed from ATP and phosphate with an Amicon Ultra 50K 0.5 mL Centrifugal Filter

(Merck-Millipore, Burlington, MA) as follows: His_6-Sec18 (100µl) was diluted with 400 µl buffer (20 mM PIPES-KOH, pH6.8, 200 mM sorbitol, 125 mM KCl, 5 mM $MgCl_2$, 2 mM DTT and 10% glycerol) and centrifuged (10,000 × g, 6 min, 4°C) to return to approximately the original volume of 100 µl. This was repeated five times, using a total of 2 ml of this buffer. To collect the final phosphate-free and ATP-free His_6-Sec18, the filter was inserted upside down in a clean collection tube and centrifuged (1000 × g, 2 min, 4°C). Protein concentration was determined by the Bradford reagent.

ATP hydrolysis reactions (20 µl) were in 0.5 ml conical plastic tubes for 10–30 min at 22°C, terminated by the addition of 20 µl of 6 mM EDTA. Proprietary solutions were added as per the manufacturer's instructions. Reactions were transferred to wells of a clear, 384-well, square well microplate (Greiner Bio-One, Kremsmunster, Austria, item 781101) and read in a BioTek EPOCH plate reader (Winooski, VT) at Ab650. All assays were performed in triplicate. Averages and standard deviations of triplicate experiments are shown, with relevant statistics (Student *t* test analysis).

ACKNOWLEDGMENTS

This work was supported by NIH grants 2R35GM118037 and GM052549. We thank Gus Lienhard, Jeremy Thorner, Alex Merz, and Axel Brunger for insightful comments.

REFERENCES

Baker RW, Jeffrey PD, Zick M, Phillips BP, Wickner WT, Hughson FM (2015). A direct role for the Sec1/Munc18-family protein Vps33 as a template for SNARE assembly. Science 349, 1111–1114.

Baker RW, Hughson FM (2015). Chaperoning SNARE assembly and disassembly. Nat Rev Mol Cell Biol 17, 465–479.

Barnard RJ, Morgan A, Burgoyne RD (1997). Stimulation of NSF ATPase activity by alpha-SNAP is required for SNARE complex disassembly and exocytosis. J Cell Biol 139, 875–883.

Barnard RJ, Morgan A, Burgoyne RD (2017). Domains of alpha-SNAP required for the stimulation of exocytosis and for N-ethylmaleimide-sensitive fusion protein (NSF) binding and activation. Mol Biol Cell 7, 693–701.

Brett CL, Plemel RL, Lobinger BT, Vignali M, Fields S, Merz AJ (2008). Efficient termination of vacuolar Rab GTPase signaling requires coordinated action by a GAP and a protein kinase. J. Cell Biol. 182, 1141–1151.

Brocker C, Kuhlee A, Getsogiannis C, Balderhaar HJK, Honscher C, Engelbrecht-Vadre S, Ungermann C, Raunser S (2012). Molecular architecture of the multisubunit homotypic fusion and vacuole protein sorting (HOPS) tethering complex. Proc Natl Acad Sci USA 109, 1991–1996.

Cheever ML, Sato TK, de Beer T, Kutateladze TG, Emr SD, Overduin M (2001). Phox domain interaction with PtdIns(3)P targets the Vam7 t-SNARE to vacuole membranes. Nat Cell Biol 3, 613–618.

Chen PS, Toribara TY, Warner H (1956). Microdetermination of phosphorus. Analytical Chem 28, 1756–1758.

Cipriano DJ, Jung J, Vivona S, Fenn TD, Brunger AT, Bryant Z (2013). Processive ATP-driven substrate disassembly by the N-ethylmaleimide-sensitive factor (NSF) molecular machine. J Biol Chem 288, 23436–23445.

Collins KM, Thorngren NL, Fratti RA, Wickner WT (2005). Sec17 and HOPS, in distinct SNARE complexes, mediate SNARE complex disruption or assembly for fusion. EMBO J 24, 1775–1786.

Fasshauer D, Sutton RBB, Brunger AT, Jahn R (1998) Conserved structural features of the synaptic fusion complex: SNARE proteins reclassified as Q- and R-SNAREs. Proc Natl Acad Sci USA 95, 15781–15786.

Fukuda R, McNew JA, Weber T, Parlati F, Engel T, Nickel W, Rothman JE, Söllner TH (2000). Functional architecture of an intracellular membrane t-SNARE. Nature 407, 198–202.

Haas A, Scheglmann D, Lazar T, Gallwitz D, Wickner W (1995). The GTPase Ypt7 of *Saccharomyces cerevisiae* is required on both partner vacuoles for the homotypic fusion step of vacuole inheritance. EMBO J 14, 5258–5270.

Haas A, Wickner W (1996). Homotypic vacuole fusion requires Sec17p (yeast α-SNAP) and Sec18p (yeast NSF). EMBO J 15, 3296–3305.

Hickey CM, Wickner W (2010). HOPS initiates vacuole docking by tethering membranes before trans-SNARE complex assembly. Mol Biol Cell 21, 2297–3405.

Hutagalung AH, Novick PJ (2011). Rab GTPases in membrane traffic and cell physiology. Physiol Rev 91, 119–149.

Jahn R, Scheller RH (2006). SNAREs- engines for membrane fusion. Nat Rev Mol Cell Biol 7, 631–643.

Jiao J, He M, Port SA, Baker RW, Xu Y, Qu H, Xiong Y, Wang Y, Jin H, Eisemann TJ, *et al.* (2018). Munc18-1 catalyzes neuronal SNARE assembly by templating SNARE association. Elife 7, e41771.

Jun Y, Wickner W (2019). Sec17 (α-SNAP) and Sec18 (NSF) restrict membrane fusion to R-SNAREs, Q-SNAREs, and SM proteins from identical compartments. Proc Natl Acad Sci USA 116, 238573–23581.

Lopes K, Orr A, Wickner W (2025). Sec18 ATP hydrolysis selectively releases Sec17 from trans-SNARE complexes, inhibiting membrane fusion. Mol Biol Cell, https://doi.org/10.1091/mbc.E24-12-0561

Ma C, Su L, Seven AB, Xu Y, Rizo J (2013). Reconstitution of the vital functions of Munc 18 and Munc 13 in neurotransmitter release. Science 339, 421–425.

Ma L, Kang Y, Jiao J, Rebane A, Cha HK, Xi Z, Qu H, Zhang Y (2016). α-SNAP enhances SNARE zippering by stabilizing the SNARE four-helix bundle. Cell Rep 15, 531–539.

Marz KE, Lauer JM, Hanson PI (2003). Defining the SNARE complex binding surface of alpha-SNAP: implications for SNARE complex disassembly. J Biol Chem 278, 27000–27008.

Mima J, Hickey CM, Xu H, Jun Y, Wickner W (2008). Reconstituted membrane fusion requires regulatory lipids, SNAREs and synergistic SNARE chaperones. EMBO J 27, 2031–2042.

Mima J, Wickner W (2009). Phosphoinositides and SNARE chaperones synergistically assemble and remodel SNARE complexes for membrane fusion. Proc Natl Acad Sci USA 106, 16191–16196.

Morgan A, Dimaline R, Burgoyne RD (1994) The ATPase activity of N-ethylmaleimide-sensitive fusion protein (NSF) is regulated by soluble NSF attachment proteins. J Biol Chem 269, 29347–29350.

Nichols BJ, Ungermann C, Pelham HRB, Wickner WT, Haas A (1997). Homotypic vacuole fusion mediated by t- and v-SNAREs. Nature 387, 199–202.

Orr A, Song H, Rusin SF, Kettenbach AN, Wickner W (2017). HOPS catalyzes the interdependent assembly of each vacuolar SNARE into a SNARE complex. Mol Biol Cell 28, 975–983

Orr A, Wickner W (2022). Sec18 supports membrane fusion by promoting Sec17 membrane association. Mol Biol Cell, 33, ar127.

Orr A, Song H, Wickner W (2022). Fusion with wild-type SNARE domains is controlled by juxtamembrane domains, transmembrane anchors, and Sec17. Mol Biol Cell 33, ar38.

Orr A, Wickner W (2024). Sec18 binds the tethering/SM complex HOPS to engage the Qc-SNARE for membrane fusion. Mol Biol Cell 35, ar71.

Prinslow EA, Stepien KP, Pan Y-Z, Xu J,Rizo J (2019). Multiple factors maintain assembled *trans*-SNARE complexes in the presence of NSF and αSNAP. Elife 8, e38880.

Rizo J, Sudhof T (2002). SNAREs and Munc18 in synaptic vesicle fusion. Nat Rev Neurosci 3, 641–653.

Schwartz ML, Merz AJ (2009). Capture and release of partially zipped trans-SNARE complexes on intact organelles. J Cell Biol. 185, 535–549.

Schwartz ML, Nickerson DP, Lobingier BT, Plemel RL, Duan M, Angers CG, Zick M, Merz AJ (2017). Sec17 (α-SNAP) and an SM-tethering complex regulate the outcome of SNARE zippering in vitro and in vivo. Elife 6, e27396.

Seals DF, Eitzen G, Margolis N, Wickner WT, Price A (2000). A Ypt/Rab effector complex containing the Sec1 homolog Vps33p is required for homotypic vacuole fusion. Proc Natl Acad Sci USA 97, 9402–9407.

Seeley S, Kato M, Margolis N, Wickner W, Eitzen G (2002). Genomic analysis of homotypic vacuole fusion. Mol Biol Cell 13, 782–794.

Shvarev D, Schoppe J, König C, Perz A, Fülbrunn N, Klontke S, Langemeyer L, Januliene E, Schnelle K, Kümmel K, *et al.* (2022). Structure of the HOPS tethering complex, a lysosomal fusion machinery. Elife 11, e80901.

Söllner T, Bennett MK, Whiteheart SW, Scheller RH, Rothman JE (1993). A protein assembly-disassembly pathway in vitro that may correspond to sequential steps of synaptic vesicle docking, activation, and fusion. Cell 75, 409–418.

Song H, Orr A, Duan M, Merz AJ, Wickner W (2017). Sec17/Sec18 act twice, enhancing membrane fusion and then disassembling cis-SNARE complexes. eLife 6, e26646.

Song H, Wickner W (2017). A short region upstream of the yeast vacuolar Qa-SNARE heptad repeats promotes membrane fusion through enhanced SNARE complex assembly. Mol Biol Cell 28, 2282–2289.

Song H, Wickner W (2019). Tethering guides fusion-competent *trans*-SNARE assembly. Proc Natl Acad Sci USA 116, 13952–13957.

Song H, Orr AS, Lee M, Harner ME, Wickner WT (2020). HOPS recognizes each SNARE, assembling ternary trans-SNARE complexes for rapid fusion upon engagement with the 4th SNARE. Elife 9, e53559.

Song H, Torng TL, Orr AS, Brunger AT, Wickner WT (2021). Sec17/Sec18 can support membrane fusion without help from complete SNARE zippering. Elife 10, e67578.

Song H, Lopes K, Orr A, Wickner W (2024). After their membrane assembly, Sec18 (NSF) and Sec17 (SNAP) promote membrane fusion. Mol Biol Cell, 35, ar150.

Sorensen JB, Wiederhold K, Müller EM, Milosevic I, Nagy G, de Groot B, Grubmüller H, Fasshauer D (2006). Sequential N to C-terminal SNARE complex assembly drives priming and fusion of secretory vesicles. EMBO J 25, 955–966.

Starai VJ, Jun Y, Wickner W (2007) Excess vacuolar SNAREs drive lysis and Rab bypass fusion, Proc. Natl. Acad. Sci. U.S.A. 104 (341) 13551–13558. https://doi.org/10.1073/pnas.0704741104

Starr ML, Hurst LR, Fratti RA (2016). Phosphatidic acid sequesters Sec18 from cis-SNARE complexes to inhibit priming. Traffic 17, 1091–1109.

Stepien KP, Rizo J (2021). Synaptotagmin-1-, and Munc13-1-dependent liposome fusion with a few neuronal SNAREs. Proc Natl Acad Sci USA. https://doi.org/10.1073/pnas2019314118

Stroupe C, Collins KM, Fratti RA, Wickner W (2006). Purification of active HOPS complex reveals its affinities for phosphoinositides and the SNARE Vam7. EMBO J 25, 1579–1589.

Stroupe C, Hickey CM, Mima J, Burfeind AS, Wickner W (2009). Minimal membrane docking requirements revealed by reconstitution of Rab GTPase-dependent membrane fusion from purified components. Proc Natl Acad Sci USA 106, 17626–17633.

Sutton RB, Fasshauer D, Jahn R, Brunger AT (1998). Crystal structure of a SNARE complex involved in synaptic exocytosis at 2.4A resolution. Nature 395, 347–353.

Thorngren N, Collins KM, Fratti RA, Wickner W, Merz AJ (2004) A soluble SNARE drives rapid docking, bypassing ATP and Sec17/18p for vacuole fusion, EMBO J 23 (297) 2765–2776. https://doi.org/10.1038/sj.emboj.7600286

Ungermann C, Nichols BJ, Pelham HR, Wickner W (1998). A vacuolar v-t-SNARE complex, the predominant form in vivo and on isolated vacuoles, is disassembled and activated for docking and fusion. J Cell Biol 140, 61–69.

Wada Y, Ohsumi Y, Anraku Y (1992). Genes for directing vacuole morphogenesis in *Saccharomyces cerevisiae*. I. isolation and characterization of two classes of *vam* mutants. J Biol Chem 267, 18665–18670.

Weninger K, Bowen ME, Chu S, Brunger AT (2003). Single-molecule studies of SNARE complex assembly reveal parallel and antiparallel configurations. Proc Natl Acad Sci USA 100, 14800–14805.

White KI, Zhao M, Choi UB, Pfueztner RA, Brunger AT (2018). Structural principles of SNARE complex recognition by the AAA+ protein NSF. Elife 10, e38888.

Wickner W, Lopes K, Song H, Rizo J, Orr A (2023). Efficient fusion requires a membrane anchor on the vacuolar Qa-SNARE. Mol Biol Cell 34, ar88.

Winter U, Chen X, Fasshauer D (2009). A conserved membrane attachment site in alpha-SNAP facilitates N-ethylmaleimide-sensitive factor (NSF)-driven SNARE complex disassembly. J Biol Chem 284, 31817–31826.

Xu H, Jun Y, Thompson J, Yates J, Wickner W (2010) HOPS prevents the disassembly of *trans*-SNARE complexes during membrane fusion. EMBO J 29, 1948–1960.

Xu H, Wickner WT (2012). N-terminal domain of vacuolar SNARE Vam7p promotes trans-SNARE complex assembly. Proc Natl Acad Sci USA 109, 17936–17941.

Zhao M, Wu S, Zhou Q, Vivona S, Capriano DJ, Cheng Y, Brunger AT (2015). Mechanistic insights into the recycling machine of the SNARE complex. Nature 518, 61–67.

Zick M, Wickner W (2013). The tethering complex HOPS catalyzes assembly of the soluble SNARE Vam7 into fusogenic *trans*-SNARE complexes. Mol Biol Cell 24, 3746–3753.

Zick M, Wickner W (2014) A distinct tethering step is vital for vacuole membrane fusion. Elife 24, 3746–3753.

Zick M, Orr A, Schwartz ML, Merz AJ, Wickner WT (2015). Sec17 can trigger fusion of trans-SNARE paired membranes without Sec18. Proc Natl Acad Sci USA, 112, E2290–E2297.

Zick M, Wickner W (2016). Improved reconstitution of yeast vacuole fusion with physiological SNARE concentrations reveals an asymmetric Rab(GTP) requirement. Mol Biol Cell 27, 2590–2597.

Zucchi PC, Zick M (2011) Membrane fusion catalyzed by a Rab, SNAREs, and SNARE chaperones is accompanied by enhanced permeability to small molecules and by lysis. Mol Biol Cell 22, 4635–4646.
