# Supplementary material for: Functional assembly of the Qc-SNARE with Sec18 and Sec17 on membranes
Source: Mol Biol Cell. 2025 Dec 19;37(1):ar5. doi: 10.1091/mbc.E25-10-0474 (PMC12766925; doi:10.1091/mbc.E25-10-0474)
Supplement: Supplementary file 2 [file reviewer_reportE25-10-0474.pdf]

Functional Assembly of the Qc-SNARE with Sec18 and Sec17 on Membranes

Amy Orr, Karina Lopes, Jerry O'Dwyer, and William Wickner

Corresponding author(s): William Wickner, Geisel School of Medicine at Dartmouth

Review Timeline:

Submission Date:	2025-10-03
Editorial Decision:	2025-10-28
Revision Received:	2025-11-04
Editorial Decision:	2025-11-08
Revision Received:	2025-11-10
Accepted:	2025-11-11

Editor-in-Chief: John Aitchison

Transaction Report:

(Note: With the exception of the correction of typographical or spelling errors that could be a source of ambiguity, letters and reports are not edited. The original formatting of letters and referee reports may not be reflected in this compilation.)

RE: Manuscript #E25-10-0474
TITLE: Functional Assembly of the Qc-SNARE with Sec18 and Sec17 on Membranes

Dear Bill:

Your manuscript has received a thorough evaluation from peer reviewers, both of whom had reasonable concerns that I hope can be readily addressed by textual changes for improved clarity and extended discussion of the relevant points raised. I look forward to reading a revised version.

Sincerely,

Liz Miller
Monitoring Editor
Molecular Biology of the Cell

Dear Dr. Wickner,

The review of your manuscript, referenced above, is now complete. The Monitoring Editor has decided that your manuscript is not acceptable for publication at this time, but may be deemed acceptable after specific revisions are made, as described in the Monitoring Editor's decision letter above and the reviewer comments below.

A reminder: Please do not contact the Monitoring Editor directly regarding your manuscript. If you have any questions regarding the review process or the decision, please contact the MBoC Editorial Office (mboc@ascb.org).

When submitting your revision include a rebuttal letter that details, point-by-point, how the Monitoring Editor's and reviewers' comments have been addressed. (The file type for this letter must be "rebuttal letter"; do not include your response to the Monitoring Editor and reviewers in a "cover letter.") Please bear in mind that your rebuttal letter will be published with your paper if it is accepted, unless you have opted out of publishing the review history.

Authors are allowed 90 days to submit a revision. If this time period is inadequate, please contact us at mboc@ascb.org.

Revised manuscripts are assigned to the original Monitoring Editor whenever possible. However, special circumstances may preclude this. Also, revised manuscripts are often sent out for re-review, usually to the original reviewers when possible. The Monitoring Editor may solicit additional reviews if it is deemed necessary to render a completely informed decision.

In preparing your revised manuscript, please follow the instruction in the Information for Authors (www.molbiolcell.org/info-for-authors). In particular, to prepare for the possible acceptance of your revised manuscript, submit final, publication-quality figures with your revision as described.

To submit the rebuttal letter, revised manuscript, and figures, use this link: Link Not Available

MBoC offers the option to publish your work with immediate open access. Open access can increase the discoverability and usability of your research. If you would like to publish your paper with immediate open access but did not select this option during initial submission, please contact the editorial office at mbc@ascb.org.

Authors of Articles and Brief Communications are also encouraged to create a short video abstract to accompany their article when it is published. These video abstracts, known as Science Sketches, are up to 2 minutes long and will be published on YouTube and then embedded in the article abstract. Science Sketch Editors on the MBoC Editorial Board will guide you as you prepare your video. Information about how to prepare and submit a video abstract is available at www.molbiolcell.org/science-sketches. Please contact mboc@ascb.org if you are interested in creating a Science Sketch.

Sign up for MBoC's new content alerts here https://tinyurl.com/3bcsyk3e

Thank you for submitting your manuscript to MBoC. We look forward to receiving your revised paper.

Sincerely,

Eric Baker
Journal Production Manager

MBoC Editorial Office
mbc@ascb.org

Reviewer #1 (Remarks to the Author):

This paper describes an interesting study of the molecular mechanisms underlying yeast vacuolar fusion suggesting that the order of events leading to assembly of the vacuolar SNARE complex is different than previously thought. SNARE complexes are formed by one R SNARE that typically resides on one of the two membranes that will fuse and by three Q SNAREs (Qa, Qb and Qc) that are commonly on the other membrane (although vacuolar membranes have all four). In addition to the SNAREs, fusion requires vacuolar lipids, the Rab protein Ypt7 and the HOPS complex, which organizes SNARE assembly, tethers the two membranes by binding to Ypt7 and acts synergistically with the SNARE disassembly machinery formed by Sec17 and Sec18. Early models assumed that three Q SNAREs form a complex that serves as an acceptor for the R SNARE. This notion was challenged by strong evidence that R and Qa come together first on the Vps33 subunit of HOPS, which acts as a template, but it was unclear how Qb and Qc are incorporated to the R-Qa complex.

The data in this paper now lead to a model whereby Qc forms a complex with Sec17, Sec18 and one of the membranes, and this complex later is integrated with an R-Qa-Qb-HOPS complex, displacing HOPS. The model is supported by a careful analysis of the factors that influence formation of the Qc-Sec17-Sec18-membrane complex, how the components of this complex as well as the other SNAREs affect ATP hydrolysis by Sec18, and how the same components influence liposome fusion in a well-established reconstitution assays. I believe that the data and the model are very intriguing, and I certainly recommend publication of the paper in MBoC, but I do have a few concerns that the authors should consider.

1. The notion that Qc first forms a complex with Sec17, Sec18 and the membrane is supported by the results presented in this paper but, considering all the available data, it seems plausible that there is more than one pathway to SNARE assembly. For instance, earlier fusion data with the reconstitution system developed by the Wickner lab suggested that Sec17 and Sec18 help with fusion but are not essential [Mima et al. (2008) EMBO J 2008, 27, 2031]. Perhaps Sec17 and Sec18 are more necessary for fusion under more stringent conditions than those used in that paper, but I am not sure that the possibility of multiple pathways of SNARE assembly, some of which do not involve the Qc-Sec17-Sec18-membrane complex, can be ruled out. I suggest that a word of caution regarding this possibility is mentioned in the discussion, or the arguments that rule out this possibility are discussed.

2. While the binding and ATP hydrolysis data of Figs. 1-5 are robust, the fusion data are not as compelling, in part because the substantial errors in the measurements make it difficult to draw firm conclusions. For instance, in lines 402-408, the authors describe that there is no statically significant difference in the results obtained with WT Sec17 and various Sec17 mutant in the absence of Sec18, whereas later in the same paragraph the effects of the same mutations on fusion in the presence of Sec18 are highlighted. This might give the impression to the readers that the mutations have differential effects in the presence but not in the absence of Sec18, whereas the data actually suggest that the FSMS and KEKE mutations inhibit fusion both in the absence and presence of Sec18 (even if there is no statistical significance without Sec18). The fusion data are still informative, but the considerable variability further limits the value of these experiments to support the model and rule out alternatives as mentioned in point 1.

3. In line 211, the authors mention a 3Sec17:Sec18 complex and provide a reference. It would be helpful to the reader to briefly introduce this complex before mentioning it in an experiment.

Josep Rizo

Reviewer #2 (Remarks to the Author):

Orr et al. present in their study their recent observations on the order of events that occur, when vacuoles fuse with each other. The authors use their established pipeline of purified proteins and proteoliposomes, and propose here that the tethering of membranes and the assembly of SNAREs is guided by the SNARE chaperones Sec17 (alpha-SNAP) and Sec18 (NSF). The authors focus in particular on the only soluble SNARE Vam7, the Qc-SNARE of the vacuolar SNARE complex. While fusion normally does not need Sec17 and Sec18 if the SNAREs are distributed asymmetrically, the authors provided previously evidence that these two chaperones have two functions. Their known role is that they bind the post-fusion SNARE complex, where three Sec17 proteins binds to the SNARE complex, and the Sec18 hexamer then uses ATP-hydrolysis to disassemble the SNAREs and primes them for fusion. This provides then SNAREs for another round of fusion. The previous work of the Wickner group suggested that the Sec17 and Sec18 chaperones can also act pre-fusion as part of the catalytic fusion machinery.

Here, the authors now show that Sec18 and Sec17 bind to a single SNARE, Vam7, and that this binding stimulates ATP-hydrolysis of Sec18. Based on this and multiple fusion experiments, the authors suggest that this is the essential function of Sec17 and Sec18 to finally deliver Vam7 (Qc) to the preassembled SNAREs and promote fusion while displacing HOPS.

This is very densely written paper, which contains a number of interesting observations. While I value the experimental effort, I have trouble following the logic and physiological impact of the study. One key problem is the presentation. The paper is written with such minimal explanations that it is hard to understand for a non-expert reader. This applies to all parts of the manuscript, including the abstract that dives deep into the nomenclature right from the beginning, and this extends through the entire manuscript. I will provide a list of recommendations and recommend a thorough revision.

1. The abstract is hard to digest. Its first line already introduces several proteins without providing any background. I would prefer names of proteins rather than Qa, Qb, Qc etc.
2. The introduction reads like a list of bullet points that are all correct, but sentences are very short and would profit from a more detailed background for the non-expert reader.
3. The titles of each paragraph are in my view not helpful and needs revision, and a similar issue applies also throughout the discussion.
4. Figure 3 and below: The authors find that Vam7 binding triggers Sec18 ATP hydrolysis. By molar ratios, how many hydrolysis events will there be for each complex? Are there several rounds of ATP hydrolysis per Vam7 molecule?
5. Results and Discussion: The paper cites a lot of work from the author's lab, yet I miss the link to the remaining literature. Here are some key questions that I find important. While it shows their previous work, it is hard to really appreciate the advances. How does this finding that there is a link between Vam7 and Sec17 ATP hydrolysis connect to our knowledge on SNARE assembly cascades? Is there evidence that Vam7 forms a selective complex with Sec17 and Sec18 on purified vacuoles - which does not include the other SNAREs?

Further issues:

1. Page 16, lane 478: Why would cells need ATP-hydrolysis by Sec18 in the absence of SNAREs at all? Could this be an in vitro artefact?
2. Page 16, lane 493: The authors state that Vam7 is in a loop-like conformation, which may explain the need for activation. I checked the AlphaFold model, but there is no evidence that the protein makes a closed conformation. It is rather a random orientation between the domains. Do they have evidence for this "loop".
3. Page 17, lane 509: This is rather a paragraph for the introduction and doesn´t help the discussion here.
4. Figures:

o Molecular weight markers are missing Gels are cropped extensively. Are full images provided in supplements?
o How many replicates were done for each experiment? What do the error bars refer to? Does MBoC still accept bar graphs or are super plots preferred?
o The model in Figure 3A does not really help to understand the assay.
o The model of the final fusion event does not reflect the size of the involved components. A more cartoon-like model may help. The authors might want to focus on SNAREs and Vps33 in their model and exclude other HOPS parts/Rabs for simplicity.

Dear Eric, and Liz, and Reviewers,

Thank you for these kind and expert reviews. We've gone through them carefully, using each suggestion to improve the manuscript. Here's a copy of the reviews with our responses in blue:

Reviewer #1 (Remarks to the Author):

This paper describes an interesting study of the molecular mechanisms underlying yeast vacuolar fusion suggesting that the order of events leading to assembly of the vacuolar SNARE complex is different than previously thought. SNARE complexes are formed by one R SNARE that typically resides on one of the two membranes that will fuse and by three Q SNAREs (Qa, Qb and Qc) that are commonly on the other membrane (although vacuolar membranes have all four). In addition to the SNAREs, fusion requires vacuolar lipids, the Rab protein Ypt7 and the HOPS complex, which organizes SNARE assembly, tethers the two membranes by binding to Ypt7 and acts synergistically with the SNARE disassembly machinery formed by Sec17 and Sec18. Early models assumed that three Q SNAREs form a complex that serves as an acceptor for the R SNARE. This notion was challenged by strong evidence that R and Qa come together first on the Vps33 subunit of HOPS, which acts as a template, but it was unclear how Qb and Qc are incorporated to the R-Qa complex.

The data in this paper now lead to a model whereby Qc forms a complex with Sec17, Sec18 and one of the membranes, and this complex later is integrated with an R-Qa-Qb-HOPS complex, displacing HOPS. The model is supported by a careful analysis of the factors that influence formation of the Qc-Sec17-Sec18-membrane complex, how the components of this complex as well as the other SNAREs affect ATP hydrolysis by Sec18, and how the same components influence liposome fusion in a well-established reconstitution assays. I believe that the data and the model are very intriguing, and I certainly recommend publication of the paper in MBoC, but I do have a few concerns that the authors should consider.

1. The notion that Qc first forms a complex with Sec17, Sec18 and the membrane is supported by the results presented in this paper but, considering all the available data, it seems plausible that there is more than one pathway to SNARE assembly. For instance, earlier fusion data with the reconstitution system developed by the Wickner lab suggested that Sec17 and Sec18 help with fusion but are not essential [Mima et al. (2008) EMBO J 2008, 27, 2031]. Perhaps Sec17 and Sec18 are more necessary for fusion under more stringent conditions than those used in that paper, but I am not sure that the possibility of multiple pathways of SNARE assembly, some of which do not involve the Qc-Sec17-Sec18-membrane complex, can be ruled out. I suggest that a

word of caution regarding this possibility is mentioned in the discussion, or the arguments that rule out this possibility are discussed. Thank you, that's correct. We've now corrected this glaring omission in a new paragraph at the beginning of the Discussion: "There are two layers of catalysis of Qc joining the SNARE complex. When there is no catalyst, i.e. when Rab/R and Rab/QaQb proteoliposomes are tethered a nonspecific agent such as polyethylene glycol (or dimeric glutathione-S-transferase fused to a lipid recognition domain such as PX or C1b), fusion has a very high EC50 for Qc and is extremely slow (Zick and Wickner, 2013; Song and Wickner, 2019). HOPS can directly bind Qc (Song et al., 2020) and lowers its EC50 for fusion (Zick and Wickner, 2013). When HOPS is limiting for fusion, Sec18 is also required to efficiently engage Qc (but not Qb) with low EC50 (Orr and Wickner, 2024). NSF/Sec18 has a direct binding site for Qc (White et al., 2018), but Qc:Sec18 association is enhanced by Sec17 through the Sec17 affinities for membranes (Zick et al., 2015; Barnard et al., 2017), Qc (Figure 1) through the Qc SNARE domain (Figure 5), and Sec18 (Söllner et al., 1993). Fusion without HOPS is blocked by Sec17/Sec18, but Sec17 and Sec18 stimulate fusion with HOPS (Mima et al., 2008). At low, physiological levels of HOPS, Qc is recruited by two tandem steps, association with Sec17 and Sec18 followed by delivery to HOPS and the zippering-blocked complex of R and Qa. Zippering is thereby enabled, turning the apolar surfaces of the R and Qa SNAREs towards each other and away from their binding sites on the Vps33 subunit of HOPS (Baker et al., 2015) to cause HOPS release."

2. While the binding and ATP hydrolysis data of Figs. 1-5 are robust, the fusion data are not as compelling, in part because the substantial errors in the measurements make it difficult to draw firm conclusions. For instance, in lines 402-408, the authors describe that there is no statically significant difference in the results obtained with WT Sec17 and various Sec17 mutant in the absence of Sec18, whereas later in the same paragraph the effects of the same mutations on fusion in the presence of Sec18 are highlighted. This might give the impression to the readers that the mutations have differential effects in the presence but not in the absence of Sec18, whereas the data actually suggest that the FSMS and KEKE mutations inhibit fusion both in the absence and presence of Sec18 (even if there is no statistical significance without Sec18). The fusion data are still informative, but the considerable variability further limits the value of these experiments to support the model and rule out alternatives as mentioned in point 1. Thank you. We had omitted a crucial point which we've now corrected. To measure the need for Sec17:Sec18 interaction for fusion, we chose a moderate level of Sec17, as now noted in the text. While high levels of Sec17 can drive fusion without Sec18, Sec 18 is also needed at moderate Sec17 levels (seen particularly clearly in (Orr and Wickner, 2022), as now cited). All comparisons between lanes which we cite as significant are either ** or ***, as shown, while differences between others we cite as

nonsignificant are indeed lost in the noise.

3. In line 211, the authors mention a 3Sec17:Sec18 complex and provide a reference. It would be helpful to the reader to briefly introduce this complex before mentioning it in an experiment. It is explicitly cited in lines 107-111 of the introduction; " A more recent model (Orr and Wickner, 2022) is that several Sec17 molecules bind reversibly to a Sec18 hexamer as a 3Sec17:Sec18 complex which stably associates with membranes by the product of the weak affinities of the N-domain apolar loop of each Sec17 for lipid. "

Josep Rizo

Thank you!

Reviewer #2 (Remarks to the Author):

Orr et al. present in their study their recent observations on the order of events that occur, when vacuoles fuse with each other. The authors use their established pipeline of purified proteins and proteoliposomes, and propose here that the tethering of membranes and the assembly of SNAREs is guided by the SNARE chaperones Sec17 (alpha-SNAP) and Sec18 (NSF). The authors focus in particular on the only soluble SNARE Vam7, the Qc-SNARE of the vacuolar SNARE complex. While fusion normally does not need Sec17 and Sec18 if the SNAREs are distributed asymmetrically, the authors provided previously evidence that these two chaperones have two functions. Their known role is that they bind the post-fusion SNARE complex, where three Sec17 proteins binds to the SNARE complex, and the Sec18 hexamer then uses ATP-hydrolysis to disassemble the SNAREs and primes them for fusion. This provides then SNAREs for another round of fusion. The previous work of the Wickner group suggested that the Sec17 and Sec18 chaperones can also act pre-fusion as part of the catalytic fusion machinery.

Here, the authors now show that Sec18 and Sec17 bind to a single SNARE, Vam7, and that this binding stimulates ATP-hydrolysis of Sec18. Based on this and multiple fusion experiments, the authors suggest that this is the essential function of Sec17 and Sec18 to finally deliver Vam7 (Qc) to the preassembled SNAREs and promote fusion while displacing HOPS.

This is very densely written paper, which contains a number of interesting observations. While I value the experimental effort, I have trouble following the logic and physiological

impact of the study. One key problem is the presentation. The paper is written with such minimal explanations that it is hard to understand for a non-expert reader. This applies to all parts of the manuscript, including the abstract that dives deep into the nomenclature right from the beginning, and this extends through the entire manuscript. I will provide a list of recommendations and recommend a thorough revision. Many thanks! We've now gone through the paper, addressing both the explicit suggestions below and also your general point that we need to strengthen the thread of narrative throughout so that the paper is more readable and easy to follow. Also, see the new paragraphs in the Discussion.

1. The abstract is hard to digest. Its first line already introduces several proteins without providing any background. I would prefer names of proteins rather than Qa, Qb, Qc etc. Thanks, we now have both at the start of the Abstract as well as in the Introduction.
2. The introduction reads like a list of bullet points that are all correct, but sentences are very short and would profit from a more detailed background for the non-expert reader. We've tried to add background (though the introduction is longer than is our custom) and to smooth the writing style. We've also added a paragraph (now the second of the Introduction) to give the "non-expert" an overview.
3. The titles of each paragraph are in my view not helpful and needs revision, and a similar issue applies also throughout the discussion. We've removed half of the paragraph titles in the Results.
4. Figure 3 and below: The authors find that Vam7 binding triggers Sec18 ATP hydrolysis. By molar ratios, how many hydrolysis events will there be for each complex? Are there several rounds of ATP hydrolysis per Vam7 molecule? Yes! In the assay tube shown in Figure 3C, lane 7, there were .03 nmoles of Sec17 and .02 nmoles of Qc, and there were 0.9 nmoles of ATP hydrolyzed. Clearly there were multiple rounds. We've now added this in the figure legend.
5. Results and Discussion: The paper cites a lot of work from the author's lab, yet I miss the link to the remaining literature. We apologize for references we missed, but at the very end of the Discussion have now added citation of Rizo's lab's work on the roles of Munc18, Munc13, and SNAP in reconstituted synaptic fusion as well as the finding that Sly 1 allows the yeast ER SNAREs to function in the presence of Sec17/Sec18. Here are some key questions that I find important. While it shows their previous work, it is hard to really appreciate the advances. How does this finding that there is a link between Vam7 and Sec17 ATP hydrolysis connect to our knowledge on SNARE assembly cascades? As we note, Sec18 ATP hydrolysis isn't needed for fusion; rather, it is a "reporter" of the ligands to which Sec18 is bound. The connection to SNARE assembly cascades is shown in Figure 6, that the same interactions governing Sec18 ATP hydrolysis are also governing fusion. Is there evidence that Vam7 forms a selective complex with Sec17 and Sec18 on purified vacuoles - which does not include the other

SNAREs? Nobody to our knowledge has ever looked for Sec18:Sec17:Qc complex on isolated vacuoles; NIH, please renew my grant so we can try!

Further issues:

1. Page 16, lane 478: Why would cells need ATP-hydrolysis by Sec18 in the absence of SNAREs at all? Could this be an in vitro artefact? This simply shows that the ATPase activity of Sec18 is sensitive to each ligand, whether a SNARE or not, and isn't meant to suggest that Sec18 does a lot of uncoupled ATP hydrolysis in the cell. We've modified this text.
2. Page 16, lane 493: The authors state that Vam7 is in a loop-like conformation, which may explain the need for activation. I checked the AlphaFold model, but there is no evidence that the protein makes a closed conformation. It is rather a random orientation between the domains. Do they have evidence for this "loop". As noted in the text, the C-terminus of the SNARE domain must face the membrane surface to be oriented for normal interactions with Sec17 and with the other SNAREs, and our data implicate the PX domain in binding the same membrane; hence the loop. We don't have physical evidence. Alpha-fold predicts that the region between the SNARE and PX domains is unstructured, consistent with, though not predictive of, such a loop.

3. Page 17, lane 509: This is rather a paragraph for the introduction and doesn´t help the discussion here. Right- this IS presented in the Introduction, and so we've deleted all but the last sentence, which adds the important point that a trans complex of HOPS, R, Qa, and Qb has been shown to be a rapid-fusion intermediate.
4. Figures:

o Molecular weight markers are missing Gels are cropped extensively. Are full images provided in supplements? We've now made it less cropped, and put in the MW markers.
o How many replicates were done for each experiment? Three, as stated for binding assays and catalytic assays in the Methods. What do the error bars refer to? Standard deviations, as stated in Methods. Does MBoC still accept bar graphs or are super plots preferred? Apparently "still" ok.
o The model in Figure 3A does not really help to understand the assay. The model visualizes each of the interactions (PX:PI3P, SNARE domain:Sec17, Sec17 loop:lipid, Sec17:Sec18) which we test with mutants or omissions in panels B and C; we hope it helps some readers to follow these tests.
o The model of the final fusion event does not reflect the size of the involved components. A more cartoon-like model may help. The authors might want to focus on SNAREs and Vps33 in their model and exclude other HOPS parts/Rabs for simplicity. Thanks for your thoughtful comment on this. Model figures must always "sail between Scylla and Charybdis" like Odysseus, where too little detail gives little information but too much detail risks implying specific atomic interactions for which there's no data. Our working model (Fig 7), like all models, is just such a set of

compromises. For example, N-domains of SNAREs aren't shown at all, we don't actually know that HOPS remains bound to the Rab on each membrane as it catalyzes initial R:Qa association (though we've shown (Torng et al., 2020) that HOPS needs to be bound to R, phosphoinositide, and Rab to catalyze soluble Q-SNARE assembly), Sec18 is actually much bigger, etc.

Thank you for this stimulating review!

RE: Manuscript #E25-10-0474R
TITLE: "Functional Assembly of the Qc-SNARE with Sec18 and Sec17 on Membranes"

Dear Bill,

I've read your revised manuscript and am satisfied that the text changes you've made address the reviewers' main points and have improved the flow. I have two final requests:
1. Please add a brief statement about the folded-back model for Vam7/Qc, stating that structural predictions suggest a disordered region that would be compatible with the presented model but that this remains to be tested directly.
2. Please add the number of replicates, that error bars are SD and the statistical tests used to the figure legends. This makes it easy for the reader to grasp the significance without having to go to the Methods. And for sure, bar plots are still fine to my mind.

Many thanks for submitting your work to MBoC. And good luck with the NIH grant!

Liz

Elizabeth Miller
Monitoring Editor
Molecular Biology of the Cell

Dear Dr. Wickner,

The review of your manuscript, referenced above, is now complete. The Monitoring Editor has decided that your manuscript requires minor revisions before it can be published in Molecular Biology of the Cell, as described in the Monitoring Editor's decision letter above and the reviewer comments (if any) below.

A reminder: Please do not contact the Monitoring Editor directly regarding your manuscript. If you have any questions regarding the review process or the decision, please contact the MBoC Editorial Office (mboc@ascb.org).

When submitting your revision include a rebuttal letter that details, point-by-point, how the Monitoring Editor's and reviewers' comments have been addressed. (The file type for this letter must be "rebuttal letter"; do not include your response to the Monitoring Editor and reviewers in a "cover letter.") Please bear in mind that your rebuttal letter will be published with your paper if it is accepted, unless you have opted out of publishing the review history.

Authors are allowed 15 days to submit a revision. If this time period is inadequate, please contact us immediately at mboc@ascb.org.

In preparing your revised manuscript, please follow the instructions in the Information for Authors (www.molbiolcell.org/info-for-authors). In particular, to prepare for the possible acceptance of your revised manuscript, submit final, publication-quality figures with your revision as described.

To submit the rebuttal letter, revised version, and figures, please use this link (please enable cookies, or cut and paste URL): Link Not Available

MBoC offers the option to publish your work with immediate open access. Open access can increase the discoverability and usability of your research. If you would like to publish your paper with immediate open access but did not select this option during initial submission, please contact us at mbc@ascb.org.

Authors of Articles and Brief Communications whose manuscripts have returned for minor revision ("revise only") are encouraged to create a short video abstract to accompany their article when it is published. These video abstracts, known as Science Sketches, are up to 2 minutes long and will be published on YouTube and then embedded in the article abstract. Science Sketch Editors on the MBoC Editorial Board will guide you as you prepare your video. Information about how to prepare and submit a video abstract is available at www.molbiolcell.org/science-sketches. Please contact mboc@ascb.org if you are interested in creating a Science Sketch.

Thank you for submitting your manuscript to MBoC. Please do not hesitate to contact this office if you have any questions.

Sign up for MBoC's new content alerts here https://tinyurl.com/3bcsyk3e

Sincerely,

Eric Baker
Journal Production Manager
MBoC Editorial Office
mbc@ascb.org

RE: Manuscript #E25-10-0474RR
TITLE: "Functional Assembly of the Qc-SNARE with Sec18 and Sec17 on Membranes"

Dear Dr. Wickner:

I am pleased to accept your manuscript for publication in Molecular Biology of the Cell.

Sincerely,
Elizabeth Miller
Monitoring Editor
Molecular Biology of the Cell

Dear Dr. Wickner:

Congratulations on the acceptance of your manuscript! Thank you for publishing your work in Molecular Biology of the Cell (MBoC).

Within 10 days, an unedited PDF of your manuscript will be published on MBoC in Press, an early-release journal version. The date your manuscript appears on this site is the official publication date.

Your copyedited and typeset manuscript will be scheduled for publication in the next available issue of MBoC. Once your paper is ready for review, our production team will notify you. In the summer of 2024, our production provider will introduce an online proofing solution, which will streamline the process of checking and validating author changes. This will result in improved typesetting quality, enhanced digital content, and a faster overall process.

We invite you to submit images related to your accepted manuscript to be considered for the journal cover.
Cover images must contain color, TIF Format
Size image at 7.4" wide x 5.85" deep
RGB and CMYK color (please submit two separate files, one in each format)
300 dpi resolution

Please also send a 1-paragraph description of the image that a general audience could understand. Include with the caption the name and institution of the individual(s) the image should be attributed to. Please provide this description in a Word file. You can send the file via email or by your favorite file transfer service to mboc@ascb.org.

Authors of Articles and Brief Communications are also encouraged to create a short video abstract to accompany their article when it is published. These video abstracts, known as Science Sketches, are up to 2 minutes long and will be published on YouTube and then embedded in the article abstract. Science Sketch Editors on the MBoC Editorial Board will guide you as you prepare your video. Information about how to prepare and submit a video abstract is available at www.molbiolcell.org/science-sketches. Please contact mboc@ascb.org if you are interested in creating a Science Sketch.

We look forward to publishing your paper in MBoC.
Sign up for MBoC's new content alerts here https://tinyurl.com/3bcsyk3e

Sincerely,

Eric Baker
Journal Production Manager
MBoC Editorial Office
mbc@ascb.org
